# *Supradapedon* revisited: geological explorations in the Triassic of southern Tanzania

**DOI:** 10.7717/peerj.4038

**Published:** 2017-11-13

**Authors:** Max C. Langer, Átila A.S. da Rosa, Felipe C. Montefeltro

**Affiliations:** 1 Laboratório de Palentologia, FFCLRP, Universidade de São Paulo, Ribeirão Preto, SP, Brazil; 2 Laboratório de Estratigrafia e Paleobiologia, Universidade Federal de Santa Maria, Santa Maria, RS, Brazil; 3 Departamento de Biologia e Zootecnia, FEIS, Universidade Estadual Paulista, Ilha Solteira, SP, Brazil

**Keywords:** Rhynchosauria, *Supradapedon*, Triassic, Selous basin, Tanzania, Tunduru, Carnian

## Abstract

The upper Triassic deposits of the Selous Basin in south Tanzania have not been prospected for fossil tetrapods since the middle of last century, when Gordon M. Stockley collected two rhynchosaur bone fragments from the so called “Tunduru beds”. Here we present the results of a field trip conducted in July 2015 to the vicinities of Tunduru and Msamara, Ruvuma Region, Tanzania, in search for similar remains. Even if unsuccessful in terms of fossil discoveries, the geological mapping conducted during the trip improved our knowledge of the deposition systems of the southern margin of the Selous Basin during the Triassic, allowing tentative correlations to its central part and to neighbouring basins. Moreover, we reviewed the fossil material previously collected by Gordon M. Stockley, confirming that the remains correspond to a valid species, *Supradapedon stockleyi*, which was incorporated into a comprehensive phylogeny of rhynchosaurs and found to represent an Hyperodapedontinae with a set of mostly plesiomorphic traits for the group. Data gathered form the revision and phylogenetic placement of *Su. stockleyi* helps understanding the acquisition of the typical dental traits of Late Triassic rhynchosaurs, corroborating the potential of hyperodapedontines as index fossils of the Carnian-earliest Norian.

## Introduction

In the course of the 1930s and 1940s, Gordon M. Stockley published a series of papers (Stockley, 1931; Stockley, 1936; Stockley, 1943; Stockley, 1946; Stockley, 1947a; Stockley, 1947b) on his geological surveys in current day Tanzania (formerly Tanganyika territory). In one of these (Stockley, 1947a), mainly dealing with coalfields around Songea explored during 1946, he mapped upper Karroo deposits to the east of that area, between the Muhuwesi and Lukimwa rivers, which he termed the “Tunduru-beds” (see Hankel, 1987). Some fossil specimens supposedly collected in that area—“from near Msamara”—were later sent to Lieuwe Boonstra, at the South African Museum, for determination. Boonstra (1953) described the remains—a tooth bearing skull portion and the proximal part of a right femur—as referred to rhynchosaurs, based on which he proposed two new species of the South American genus *Scaphonyx*, respectively *Sc. stockleyi* and *Sc*. *africanus*, which were very seldom mentioned in more recent studies.

In his review of rhynchosaur evolution, Chatterjee (1980) considered *Scaphonyx africanus* a junior synonym of *Sc. stockleyi* and erected a new genus—*Supradapedon*—to accommodate that species. Later, Hunt & Lucas (1991) proposed a *nomen dubium* status for *Sc. africanus* and assigned *Su. stockleyi* to the Hyperodapedontinae (see also Benton, 1983). More recently, Langer et al. (2000) revised the inclusivity of the genus *Hyperodapedon*, proposing that it encompassed most Late Triassic rhynchosaurs, including *Su. stockleyi* (see also Mukherjee & Ray, 2014), but did not address the validity of that species. Such attribution was then used to stratigraphically correlate the strata bearing *Su. stockleyi* in southern Tanzania to the *Hyperodapedon*-rich Carnian Stage (Late Triassic) of other parts of Pangaea (Lucas & Heckert, 2002; Langer, 2005).

**Figure 1:**
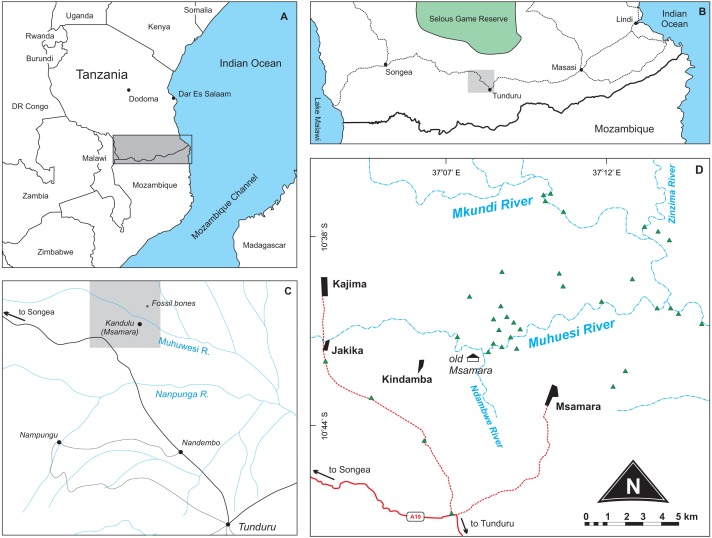
Location maps of the study area. (A) Map of southeast Africa showing the area highlighted in B; (B) Map south Tanzania showing the location (grey square) of the study area; (C) Map of the study area redrawn from Stockley (1947a) showing the area highlighted in D; (D) Main area of prospection, with surveyed sites marked as green triangles. Further described sites to the south are shown in Fig. 2.

**Figure 2:**
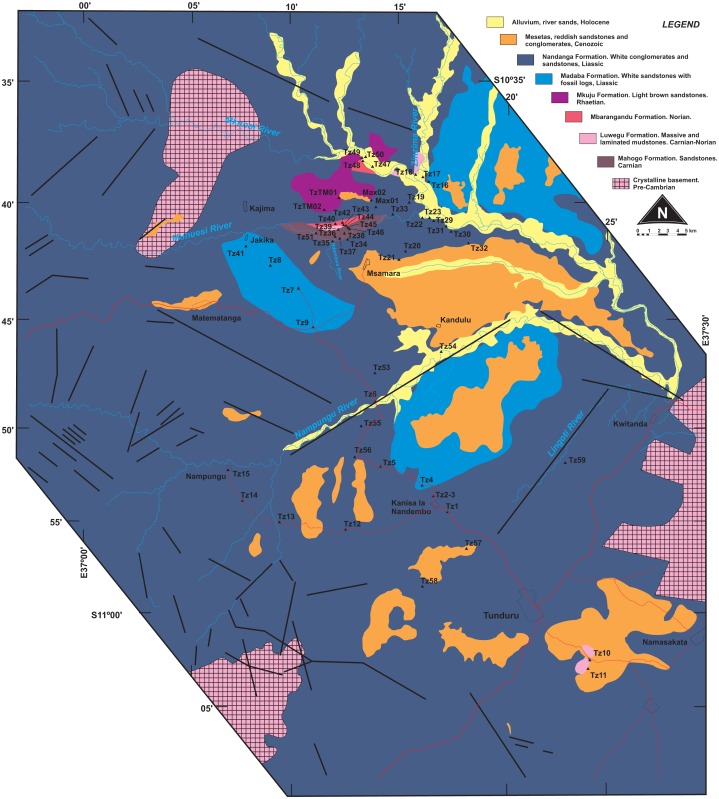
Surface geology map of the study area (part of Quarter Degree Sheets 301, 302, 313, 314 from the Geological Survey of Tanzania). Surveyed sites marked as black triangles.

Stockley (1947a; fig. 1) presented a surface geology map where a “fossil bones” symbol is seen about 2.5 miles to the north of the Kundulu (Msamara) village. Gathering information from local inhabitants during the 2015 fieldwork revealed that Kundulu was the name given to a hunting site near the old Msamara village, whereas the village itself was moved to about five kilometres southeast of its original location. We assumed the “fossil bones” symbol as meant to indicate the site where the rhynchosaur material described by Boonstra (1953) was found. Following that premise, we prospected the area during July 2015, in search for both Late Triassic fossils and their bearing rocks. More than sixty outcrops were mapped (Figs. 1–2), mostly representing upper Karroo deposits (see “Geological settings” below), but only fossilized logs and Holocene subfossils were found, the former mostly scattered on the surface; i.e., lacking geological context. With no Triassic vertebrates found, it remains uncertain if the material described by Boonstra (1953) was collected in the site mapped by Stockley (1947a), and its provenance cannot be confirmed at the moment. Nonetheless, the new geological data presented below, along with a more in deep evaluation of the anatomy and affinities of *Supradapedon stockleyi*, provides an updated framework onto which the Late Triassic of south Tanzania and their vertebrate fossils can be more carefully contextualized.

## Material and Methods

### Phylogenetic analysis

To test the affinities of *Supradapedon stockleyi* among rhynchosaurs, a phylogenetic analysis was conducted based on a data matrix modified from Schultz, Langer & Montefeltro (2016). The data-matrix was modified to expand the Hyperodapedontinae sample, as the original matrix included all species of *Hyperodapedon* collapsed into the terminal taxon *Hyperodapedon* spp. Here, we included all putative species of *Hyperodapedon* (i.e., *H. gordoni, H. huenei, H. huxleyi, H. mariensis, H. sanjuanensis*, *H. tikiensis*, and *Su. stockleyi*) as terminal taxa, as well as unnamed forms previously attributed to that genus (Zimbabwe, Wyoming, and Nova Scotia forms). We also added characters to the original matrix of Schultz, Langer & Montefeltro (2016): six (numbers 113–118) from Mukherjee & Ray (2014) and five (numbers 119–123) based on newly recognized variations within our ingroup (see additional information on sampled characters and taxa in the Supplemental Information 1). The single non-cranial character scored for *Su. stockleyi* (number 94) was based on the holotypic femur of *Scaphonyx africanus* (SAM-PK-11705). The final matrix, composed of 25 taxa and 123 characters, was analysed using the software TNT *version* 1.5 (Goloboff, Farris & Nixon, 2008) under the implicit enumeration algorithm, having *Protorosaurus speneri* as the primary outgroup, collapse of “zero-length” branches (minimum length is = 0), and with multistate characters 70 and 76 treated as additive.

### Field work permit

The field work and fossil collection was granted by Excavation/Collector Licence No: 21/2014/2015 from the Ministry of Natural Resources and Tourism, The United Republic of Tanzania.

## Results

### Geological settings

**Figure 3:**
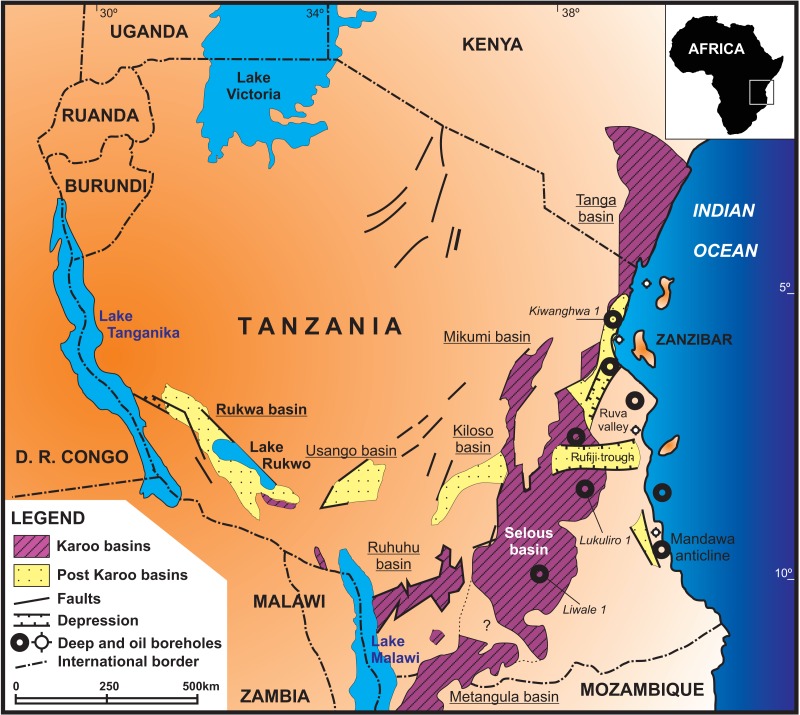
Location and tectonostratigraphic context of the Selous Basin, its subdivisions and correlation with neighbouring Karoo and post-Karoo basins. Based on a regional geology map from the Tanzania Geological Survey, with information from Verniers et al. (1989) and Catuneanu et al. (2005).

The Selous (or Luwegu) Basin (Fig. 3) is a NE–SW depocenter that connects with the Metangula (or Ruvuma) Basin in Mozambique, extending northwards in a “Y-shaped” striking graben structure (Hankel, 1987; Catuneanu et al., 2005) as the Mikumi Basin to the west and the Rufiji trough and Tanga Basin to the east (Kreuser et al., 1990). Oil and uranium prospection in the 1980’s led to geological mapping, seismic acquisition and deep borehole perforation, like the “Liwale 1” borehole in the central part of the basin (Kreuser et al., 1990; Wopfner, 1994; Thornton, 2014).

**Figure 4:**
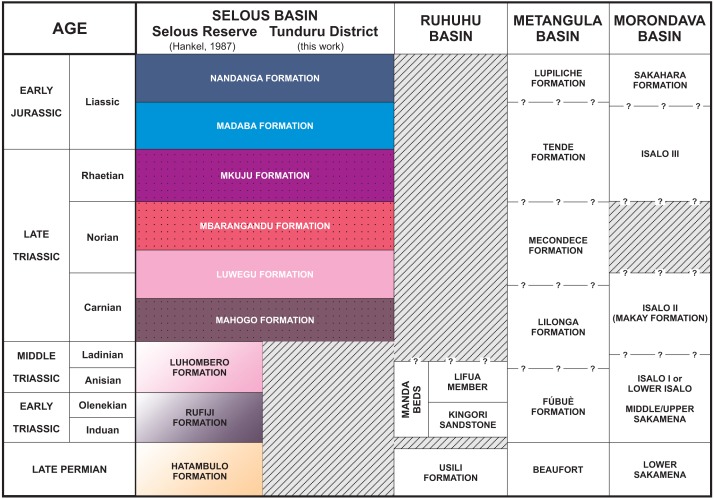
Stratigraphic chart of the Selous Basin and tentative correlation with neighbouring basins (compiled from Hankel, 1987; Piqué et al., 1999; Catuneanu et al., 2005; Araújo, Castanhinha & Junior, 2012).

Our study area comprises a polygon that covers the town of Tunduru, Ruvuma Region, southern Tanzania, and several villages in the vicinities of A19 road (Fig. 2). Geological observations on this area allowed correlation of natural (river banks, erosional gullies) and artificial (road cuts, sand and gravel mines) outcrops to Hankel’s (1987) lithostratigraphic scheme (Fig. 4). Over 60 outcrops were described in the area, in which three depositional successions are the most important features: (1) probably Triassic reddish mudstones, sandstones and conglomerates (red beds); (2) probably Jurassic whitish sandstones, with localized levels of gray or red mudstones, and allochthonous silicified logs; and (3) red sandstones and conglomerates, in flat-topped hills of Cenozoic age.

**Figure 5:**
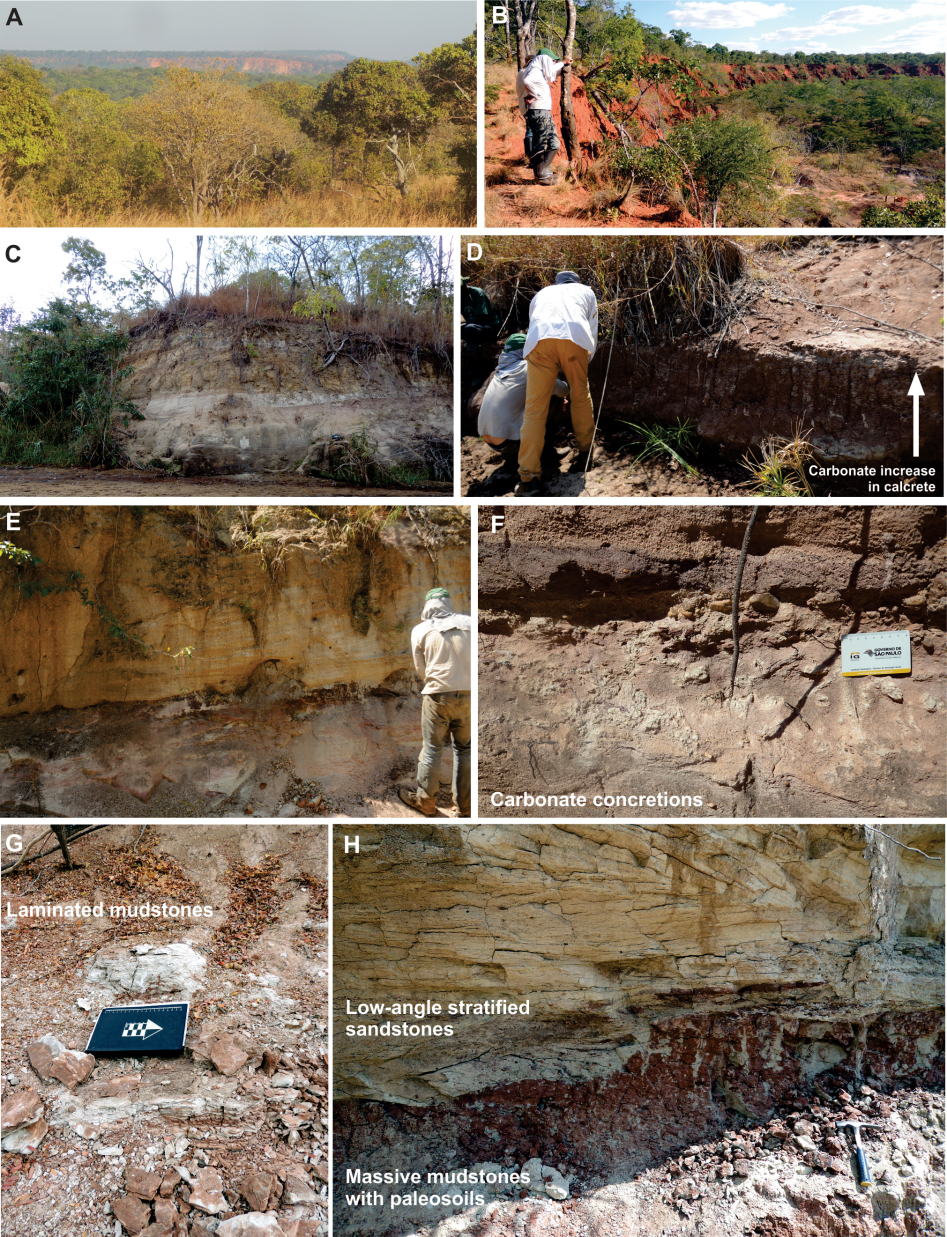
(A) SW view of a meseta on the background, taken from the A19 road; (B) amphitheatre outcrop (Tz 21), preserving Cenozoic reddish deposits over older sequences; (C) outcrop along the Muhuesi river (Tz 36), with cross trough stratified sandstones of the Mahogo Formation; (D) river bank outcrop north of the Muhuesi river (Tz 44) with calcretes in the Mbaragandu Formation; (E) outcrop along the Lukoswe river (Tz 40) with stratified sandstones (Mkuju Formation) over massive mudstones (Mbaragandu Formation); (F) carbonate concretions in the Mbaragandu Formation (Tz 43); (G) outcrop at the Zinzima river (Tz 18), with laminated mudstones, with climbing ripples; (H) outcrop along the Mkundi river (Tz48), with low-angle stratified sandstones of the Mkuju Formation over the massive mudstones of the Luwegu Formation.

The flat-topped hills (or mesetas) are the most prominent topographic/geomorphological features in the area (Fig. 5A), with the following lithologies: (a) massive, quartzose orthoconglomerates, with subrounded pebbles up to 10 cm in diameter and ferruginous matrix; (b) reddish, conglomeratic sandstones, massive or cross-bedded; and (c) massive, reddish medium to coarse sandstones, with a hard ferruginous matrix. As these lithologies play an important role in the maintenance of the relief, pluvial and alluvial erosion acted together in the formation of walls and amphitheatres (Fig. 5B), seen from distance, and preserving older sequences.

Whitish sandstones, with localized levels of gray or red mudstones, and allochthonous silicified logs were described in several outcrops. These correspond to the Nandanga and Madaba formations of Hankel (1987). The Nandanga Formation is widespread all over the mapped area, recording coarse sandstones and conglomerates, whereas the Madaba Formation occurs at some outcrops (Tz 6, 7, 8, 20, 23, 29, 31, 33, 34, 35, 38), with medium to coarse sandstones and layers and/or intraclasts of gray fine-grained lithologies (sandstones and mudstones). Decimetric pieces of silicified logs are recorded in these lithologies, frequently rounded and up to 30 cm long. Cross-trough stratifications suggest braided streams with an overall NE paleoflow. This lithofaciological description and the abundance of fossil logs of seemingly the same kind (no other fossils were found) are the base to correlate these rocks to the Nandanga and Madaba formations of the lithostratigraphic scheme of the norther part of the basin (Hankel, 1987).

Red beds around Tunduru are well exposed near Namasakata (Tz 10, 11), along and to the south side of the Muhuesi River (Tz 21, 32, 36, 37, 40), along and to the north side of the Mkundi River (Tz 18, 47, 48, 49, 50). These lithologies are represented by the intercalation of fine sandstones and siltstones (Tz 18), with climbing ripples (Tz 18, 21), or coarse, quartzo-feldspatic sandstones, with fine-grained layers and/or intraclasts (Tz 36, 37). Cross trough stratifications and climbing ripples suggest a SW paleoflow, in low energy streams, that preserved high sinuosity channels and floodplain fines. The floodplain deposits record temporary lakes, and pedogenetic features, such as carbonate concretions. These rocks correspond to the Mahogo, Luwegu, Mbarangandu and Mkuju formations of Hankel (1987).

Hankel (1987) proposed a lithostratigraphic scheme for the northern portion of the Selous Basin (Fig. 4), including two packages of red beds deposited together (Madaba and Mkuju formations) and the Luwegu Formation as part of a Carnian-Norian deposition cycle. Conglomerates of the Nandanga Formation, containing fossil wood, represent the top of the Triassic/Jurassic sequence in the Tunduru area, which is below the Cenozoic deposits. Given its widespread distribution (Fig. 2), the Nandanga Formation probably corresponds to most of the “Tunduru beds” of Stockley (1947a), as also proposed by Hankel (1987). Based mainly on macro- and microfloristic assemblages, Hankel (1987) suggested a Carnian age for the braided deposits from the Mahogo Formation, a late Carnian to early Norian age for the fluviolacustrine Luwegu Fomation, and a late Norian age for the braided deposits form the Mbarangandu Formation. A second red-bed depositional cycle is composed of the Rhaetian braided deposits from the Mkuju Formation, and the “Liassic” meandering deposits of the Madaba Formation.

In fact, only upper Triassic and lower Jurassic rocks are present in the southeastern portion of the Selous Basin, as the Hatambulo (late Permian), Rufiji (“Scythian”), and Luhombero (Middle Triassic) were not recognized at the studied area. It is not clear, however, if those lithologies could occur in other (sub)basins south or southwest from Tunduru. For example, in Mbesa, 45 km SW from Tunduru, there are conglomerates and coarse sandstones, dipping north-westwards, locally known as “Karoo deposits” (Geol. Abraham Nkya, Tunduru office of the Ministry of Energy and Mines, personal communication). It is uncertain to which formation these deposits belong, if they were tectonically tilted, or represent a proximal facies in the border of the basin, but this is beyond the scope of this work.

**Figure 6:**
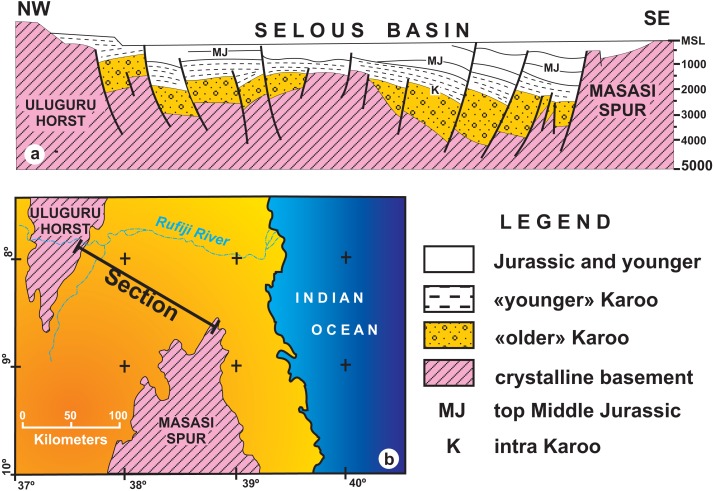
(A) Geological interpretation of a low–resolution seismic section from the Selous Basin; (B) Map showing the transect reproduced in A (redrawn form Wopfner, 1994). Note the thickness and regional unconformity of “older” Karoo (Hatambulo, Rufiji and Luhombero formations from Hankel, 1987) and “younger” Karoo deposits (Mahogo, Luwegu and Mbaragandu formations).

The successive depositional cycles studied in the Tunduru area can be related to second order events, such as the breakup and rifting of the African-Indian plates and formation of the Malagasy Rift (Rosendahl, 1987). These events are recorded as regional unconformities, displayed in seismic sections. An intra-Karoo unconformity (Fig. 6) comprises landscape formation in the Triassic (King, 1978; Wopfner, 1994; Wopfner, 2002) or late Permian-Early Triassic (Miller, 1997). In fact, there is an overall discrepancy on the Permo-Triassic record in the basins formed on the opening of the Malagasy Rift (Fig. 4). For example, the central portion Selous Basin, as well as the Metangula (Araújo, Castanhinha & Junior, 2012) and Morondava (Piqué et al., 1999) basins, records a deposition spanning from the late Permian to the Early Jurassic, but only Late Triassic to Early Jurassic sequences are recorded in its southern portion, whereas the Ruhuhu Basin has a late Permian to Middle Triassic infilling (Catuneanu et al., 2005; Nesbitt et al., 2017). All these basins were somehow related on the fragmentation process of Gondwana, but further details are still inconclusive.

Despite being an outdated, low resolution seismic section, the geophysical data taken at the northern part of the Selous Basin (Fig. 6), in which the total thickness is in the order of hundreds of meters (Wopfner, 1994), severely contrasts with the thicknesses (thousands of meters) proposed by Hankel (1987). For instance, according to Wopfner (1994), “older” Karoo deposits average a thousand meters near the Uluguru Horst, reaching a maximum of 1,500 m near the Masasi Spur. Instead, Hankel (1987) measured a total thickness of up to 4,400 m for the Hatambulo (late Permian), Rufiji (Early Triassic), and Luhombero (Middle Triassic) together, and up to 1,550 m for the Mahogo (300 m), Luwegu, (200 m), Mbarangandu (250 m), Mkuju (200 m), Madaba (300 m), and Nandanga (300 m). Differences in thickness from the margin to the centre of the basin may be expected due to a more subsident depocenter than the basin borders, but the differences from the “older” Karoo (4,400 m for Hankel, 1987; 1,500 m for Wopfner, 1994) to the “younger” Karoo (1,550 m for Hankel, 1987; <1,000 m for Wopfner, 1994) are still to be comprehensively understood.

As the most pervasive lithology in the study area, the general correspondence of the Nandanga Formation to most of the “Tunduru beds” is feasible. Yet the “Tunduru beds” of Stockley (1947a) encompasses more disparate lithologies (Figs. 2 and 4). Among these, the inferred time range of hyperodapedontine rhynchosaurs in other basins (see below) and the stratigraphic correlation scheme proposed here (Fig. 4) indicate that the beds that yielded the rhynchosaur fossils described by Boonstra (1953) are most probably related to the lower units of the sequence, which have a more patchy surface distribution in the study area (Fig. 2). Accordingly, we infer that the Tunduru rhynchosaurs fossils came from either the Luwegu or Mahogo formations, which represent the Carnian deposits in the area. Most probably the later, given its surface mapping (Fig. 2).

### Systematic palaeontology

**Table:** 

Rhynchosauria Osborn, 1903 (*sensu* Dilkes, 1998).
Rhynchosauridae Cope, 1870 (*sensu* Dilkes, 1998).
Hyperodapedontidae Lydekker, 1885 (*sensu* Langer & Schultz, 2000a).
Hyperodapedontinae (Chatterjee, 1969 *nom. trans. ex* Lydekker, 1985 *sensu* Langer & Schultz, 2000a)
*Supradapedon stockleyi* (Boonstra, 1953 *gen*. Chatterjee, 1980)
Figs. 7–9
1953 *Scaphonyx africanus* Boonstra, p. 4; Fig. 2.
1980 *Supradapedon stockleyi* Chatterjee, pp. 58,59; plate I.E.
1983 *Supradapedon stockleyi* Benton, p. 712
1991 *Supradapedon stockleyi* Hunt & Lucas, p. 933
1991 *Scaphonyx africanus* Hunt & Lucas, p. 933
2000b *Supradapedon stockleyi* Langer & Schultz, p. 252
2000 *Hyperodapedon stockleyi* Langer et al. p. 117
2008 *Supradapedon stockleyi* Hone & Benton, p. 107
2014 *Hyperodapedon stockleyi* Mukherjee & Ray, pp. 10, 27, 28, 30, 32, 33, table 2

*Holoype*. SAM-PK-11704, fragment from the left side of the skull, composed of a nearly complete maxilla and partial bones (palatine, jugal, ectopterygoid) surrounding the maxillary tooth plate.

**Figure 7:**
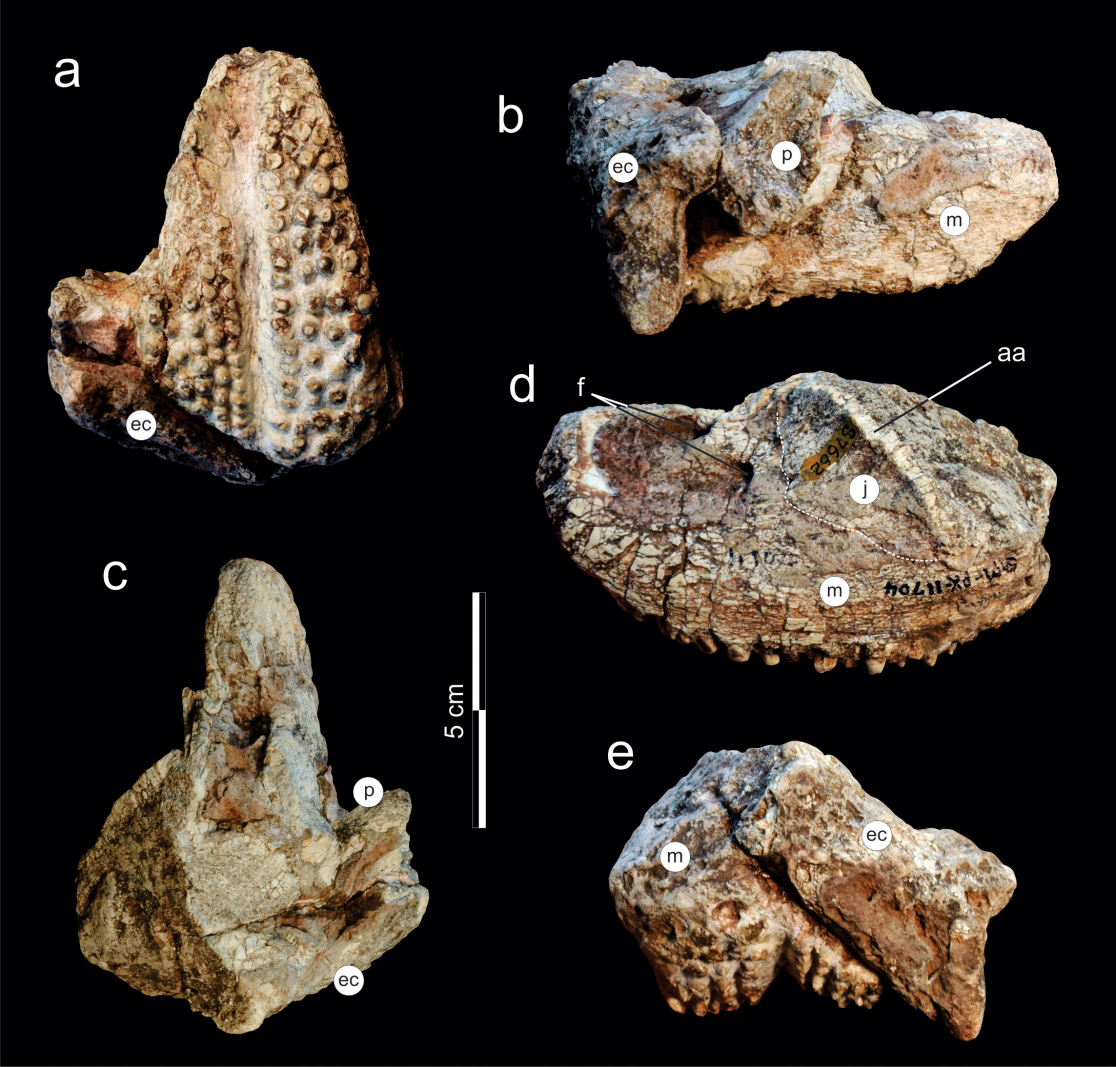
*Supradapedon stockleyi*, SAM-PK-11704 (holotype), skull fragment in (A) ventral; (B) medial; (C) dorsal; (D) lateral; and (E) caudal views. Abbreviations: aa, *anguli oris* crest; ec, ectopterygoid; f, foramen; j, jugal; m, maxilla; p, palatine.

**Figure 8:**
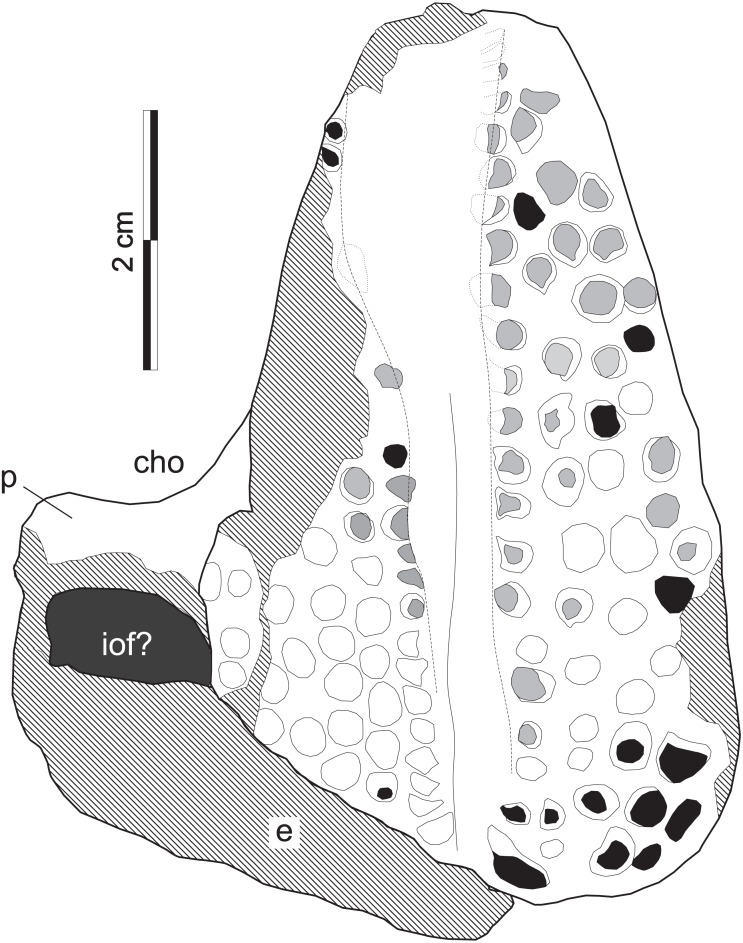
*Supradapedon stockleyi*, SAM-PK-11705 (holotype), interpretative drawing in ventral view. Abbreviations: cho, choana; e, ectopterygoid; iof, infraorbital fenestra; p, palatine. Hatched areas indicate lost bone surface. Worn-out teeth marked in grey; broken-off teeth marked in black.

*Probable referred material*. SAM-PK-11705 (holotype of *Scaphonyx africanus* Boonstra, 1953) proximal portion of a right femur. That specimen bears an internal trochanter separated from the femoral head, which is synapomorphic for Hyperodapedontinae, providing a phylogenetic signal that agrees with that of the cranial and dental characters of SAM-PK-11704. Therefore, we for the moment agree with Chatterjee (1980), who considered *Sc. africanus* Boonstra, 1953, a junior synonym of *Su. stockleyi* (Boonstra, 1953, gen. Chatterjee, 1980).

*Type locality*. “From near Msamara, Tunduru district” (Boonstra, 1953). Based on the “fossil bones” sign in Stockley (1947a, fig. 1) this should be about 4 km N-NE of the old village of Msamara. Plotted to a current cartographic base, this corresponds to a spot located around 10°40′S/37°09′E. The entire area was surveyed during the 2015 expedition, but no outcrops were found in that spot and the precise location of the type-locality remains unknown.

*Type horizon*. Based on the surface geology recognition of the Msamara area (Fig. 2), the spot marked in Stockley (1947a, fig. 1) would correspond to either the Mahogo or Nadanga formations. As such, given its inferred Carnian age, the more likely type-horizon corresponds to the sandstones of the Mahogo Formation.

*Diagnosis*. The *Supradapedon stockleyi* holotype differs from the type-specimens of all other species of Hyperodapedontinae, as well as from all specimens of the group known by the authors, based on the unique combination of a medial maxillary “tooth-bearing area” (TBA) that is narrower than the lateral, but bears more (6 vs 4) longitudinal tooth rows. Yet this condition represents a mixture of plesiomorphic and apomorphic traits that vary along the dental evolutionary pathways identified for hyperodapedontines (see below). Accordingly, it probably does not correspond to an autapomorphic condition and might equally be found in taxa/specimens yet to be discovered. Nonetheless, topotypic principles also support the status of *Su. stockleyi* as a valid taxon, and this is endorsed here. In fact, the only other specimen supposedly found together with the holotype of *Su.* *stockleyi* and tentatively assigned to that species (SAM-PK-11705; i.e., the holotype of *Sc. africanus*, coined in the same paper as *Sc*. *stockleyi*) also bears a unique condition among hyperodapedontines, i.e., femoral head with dorsoventral and craniocaudal axes of similar size.

### Description

The holotype of *Supradapedon stockleyi*, SAM-PK-11704, corresponds to a fragment from the left side of the skull, including most of the maxilla, and parts of the jugal, palatine, and ectopterygoid (Figs. 7–8). The elements other than the maxilla are very badly preserved, lacking most of their original external bone surface. This hampers recognizing sutures among bones, so that the identification of each element is mostly based on their relative positions compared to that of other Hyperodapedontinae (Chatterjee, 1974; Benton, 1983; Langer & Schultz, 2000a; Montefeltro, Langer & Schultz, 2010).

**Figure 9:**
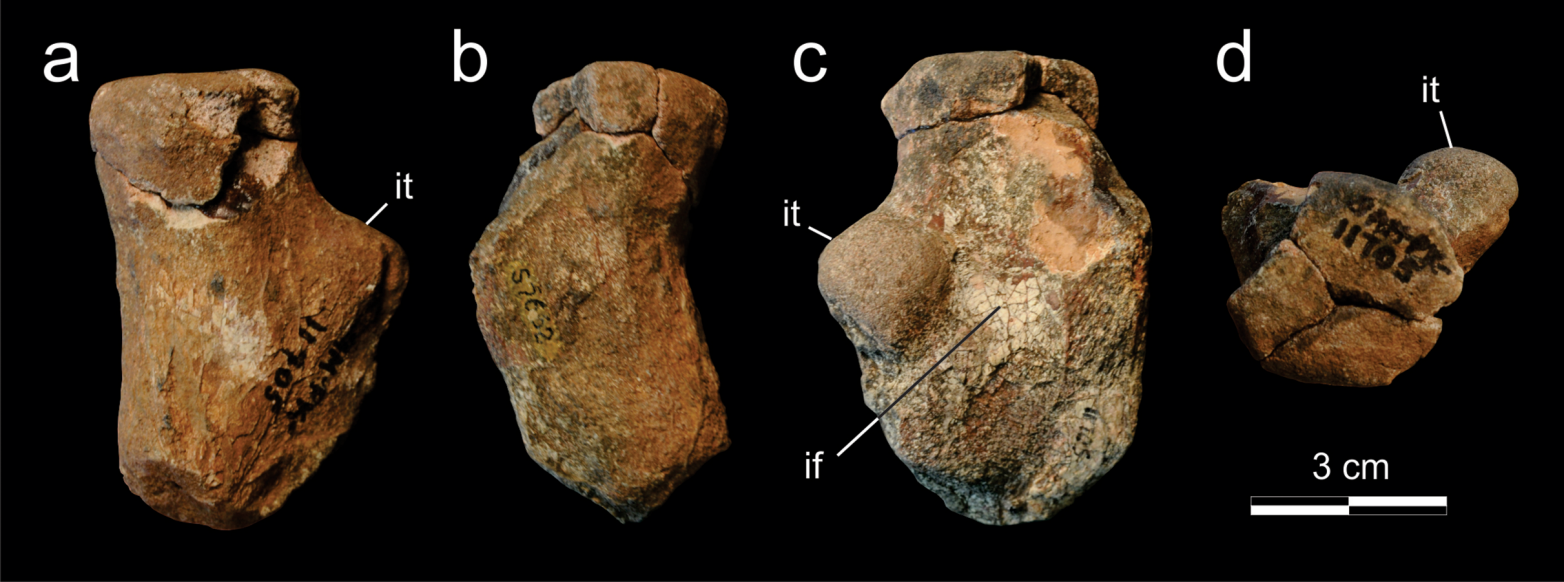
*Supradapedon stockleyi*, SAM-PK-11705, proximal portion of the right femur in a, dorsal; b, caudal; c, ventral; and d, proximal views. Abbreviations: if, intertrochanteric fossa; it, internal trochanter.

The jugal is better seen in lateral view (Fig. 7D), represented by an eroded *anguli oris* crest and the small lobe of the bone positioned rostroventral to that. The *anguli oris* crest is caudoventrally to rostrodorsally oriented, extending across the rostral process of the jugal. Considering the preserved portion of the crest, it was probably limited to the jugal bone, not extending onto the maxilla. The jugal dorsally overlaps the lateral margin of the caudal half of the maxilla. Yet it is unclear how far the jugal spreads medially over the maxilla, due the poor preservation of the area. As such, it is not clear how far the jugal extended medially to reach the ectopterygoid, bracing the maxillary tooth plate caudally. The palatine extends medially from the caudal third of the medial margin of the maxillary tooth plate. It forms a rostrally concave surface, which represents the caudal margin of the choana (Fig. 8). The medial margin of the palatine is not preserved and the caudal portion has lost most of its external bone surface. As a result, its articulation to other palatal bones and participation in the inferior orbital foramen cannot be clearly identified (Fig. 8). Caudal to that area, an element also lacking most of its bone surface was identified as part of the ectopterygoid, the boundaries of which are equally elusive. It forms a broad vertical wall covering the medial half of the caudal margin of the maxilla, which corresponds to the “posterior surface” of the “dorsolateral process” of Langer & Schultz (2000a). A short process corresponding to the “anterior surface” of the “dorsolateral process” (Langer & Schultz, 2000a) extends medially from the dorsal half of the vertical wall. It likely forms part of the orbital cavity floor and the caudal margin of the inferior orbital foramen, but these boundaries cannot be clearly observed (Fig. 8). The “posteroventral process” of the ectopterygoid (Langer & Schultz, 2000a) was not preserved.

As typical of hyperodapedontine rhynchosaurs, the maxilla of *Supradapedon stockleyi* is a deep, rostrocaudally elongated bone, with the convex, subtriangular (rostrally tapering) occlusal surface forming a tooth plate. The rostral ascending process that usually projects dorsally from the tooth-bearing area rostral to the lachrymal to overlap the premaxilla (e.g., Benton, 1983) is broken off. As recognized by Boonstra (1953), the lateral surface of the maxilla, rostral to the jugal articulation, hosts two nutrient foramina that may correspond to enlarged versions of the “lateral alveolar foramina” of Benton (1983), as also seen in *Hyperodapedon tikensis* (Mukherjee & Ray, 2014) and some South American specimens attributed to that genus (MACN-Pv 1885, BSPG 19.4; MCP 1963, PVL 2080, PVSJ 679). The occlusal surface of the maxilla is 108 mm long and reaches 58 mm in maximal lateromedial breadth. Compared to the *Hyperodapedon* sp. sample at the UFRGS collection (Horn et al., 2015), this represents a relatively large animal, with a skull nearly 20 cm long, i.e., about 70% the size estimated for the largest individual housed in that collection.

Tooth shape and distribution are taxonomically informative for rhynchosaurs (Chatterjee, 1980; Langer et al., 2000). The occlusal surface of the maxilla in SAM-PK-11704 bears a well-developed, single groove that sets the medial and lateral cushion-shaped tooth bearing areas (TBAs) apart. Here, we employ the nomenclature of longitudinal tooth rows in the maxilla proposed by Chatterjee (1974), in which the lateral rows to the main groove are labelled as L1, L2, etc., and those medial to the main groove as M1, M2, etc. The shape of the groove is in part formed by the occlusion of the blade-like dentary (Benton, 1984) during the animal’s life, as shown by some nearly worn-out tooth elements (ex: rostral teeth in M1 and L1). The groove is narrower in the caudal half of the maxilla, but expands medially at the rostral half, as in some *Hyperodapedon* specimens (Benton, 1983; Langer & Schultz, 2000a; UFRGS-PV-0408T, 0149T; PVSJ 679). The medial TBA is broken at its caudomedial corner and its surface is worn-out for most of the medial margin (Fig. 8). In average, as well as at the point of maximum breadth of the maxilla (27 vs 25.5 mm), the lateral TBA is slightly broader than the medial (Langer & Schultz, 2000a). The lateral TBA bears a maximum of four longitudinal tooth rows (L1 to L4), whereas a maximum of six rows are present on the medial TBA (M1 to M6). In the medial TBA, the single M6 tooth is displaced dorsally and inserted at the boundary between the occlusal and lingual surfaces of the maxilla. In addition, the caudal edge of the TBAs are rounded and the lateralmost and medialmost teeth are not directed strictly ventrally, but latero- and medioventrally respectively. Most teeth are conical, but those of M1 and L1 are of the “pyramidal” type (Benton, 1984); i.e., with an angular rather than rounded cross section. These teeth are roughly of the same size of most conical teeth, so that no substantial variation in tooth size is seen in the plate. The tooth bearing occlusal surface forms nearly a right angled corner to the lateral surface of the maxilla. Although partially broken, this also seems to be the case for its medial counterpart.

The holotype of *Scaphonyx africanus* Boonstra, 1953, SAM-PK-11705, is the only putative record of the postcranial anatomy for *Supradapedon stockleyi* (Fig. 9). This specimen consists of a proximal portion of the right femur (7.5 cm of preserved length), including the femoral head and the proximal part of the shaft. Most of the external surface of the fragment is worn-out and the articulation area is severely damaged by breakages, so that few information can be extracted from this specimen. The preserved portion of the shaft suggests the presence of a constriction in the transition from the femoral head, as usually present in rhynchosaurids (Montefeltro et al., 2013). The original shape of the proximal articulation area is obscured by breakages, but its surface is flat and the dorsoventral and craniocaudal axes are similar in size, which represents an unique condition among hyperodapedontines. The proximal most portion of the internal trochanter suggest a well-developed, robust and roughly spherical structure. Still, the presence of a crest-shaped distal extension and its continuity to the ventral adductor crest cannot be confirmed. The internal trochanter is separated from the proximal articular surface by an excavation, as frequent in hyperodapedontines (Montefeltro et al., 2013). The internal trochanter forms the cranial margin of the intertrochanteric fossa, which corresponds to a faint concavity at the ventral surface of femoral head. This is more developed proximodistally than craniocaudally, as seen in the holotype of *Teyumbaita sulcognathus* (Montefeltro et al., 2013). The intertrochanteric fossa is bounded caudally by a weak crest, but the surface of that crest is worn-out and the possible insertion area for part of the *M. puboischiofemoralis externus* is not preserved.

**Figure 10:**
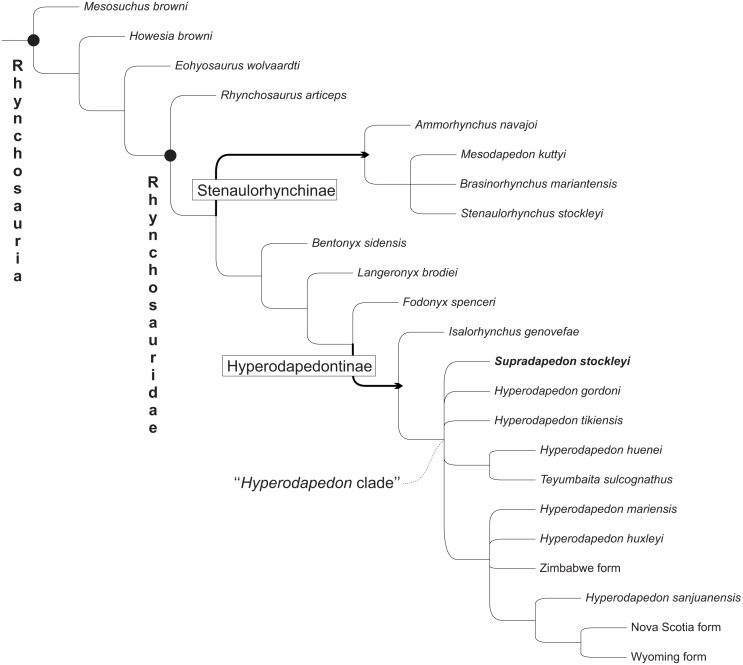
Strict consensus of the 11 most parsimonious trees found in the present analysis, not showing *Protorosaurus speneri* and *Prolacerta browni*. Black circles indicate node-based taxa; thick arrows indicate branch-based taxa.

## Discussion

### Affinities of *Supradapedon*

Our phylogenetic analysis resulted in 11 most parsimonious trees (MPTs) of 216 steps, that mostly agree with the results of Schultz, Langer & Montefeltro (2016), the strict consensus of which is depicted in Fig. 10 (see the Supplemental Information 1 for full list of synapomorphies, as well as “Bremer support” and bootstrap resampling values for each clade). *Supradapedon stockleyi* nests within the Hyperodapedontinae, defined as the most inclusive clade to include *Hyperodapedon gordoni*, but not *Fodonyx spenceri* (modified from Langer & Schultz, 2000a; Langer & Schultz, 2000b), sharing the following synapomorphies with other members of the clade: an *anguli oris* crest that extends to the rostral process of the jugal (ch. 14; state “2”); a jugal that extensively overlaps the maxilla laterally (ch. 16; state “1”); a lateral maxillary TBA that is broader than the medial (ch. 67; state “1”), “cushion-shaped” (i.e., broader than deep; ch. 68; state “1”), and formed by more than one clear longitudinal tooth row (ch. 69; state “1”) (the increase of tooth rows is also present in the medial TBA (ch. 70), as well as generally in the rostral part of the bone (ch. 71), representing traits that place *Su. stockleyi* closer to *Teyumbaita* and *Hyperodapedon* than to *Isalorhynchus*); a reduced number of maxillary lingual teeth (restricted in *Su. stockleyi* to the interface between the lingual and occlusal surfaces of the bone; ch. 73; state “0”); and maxillary pyramidal teeth (ch. 74; state “1”). In addition, based on SAM-PK-11705, *Su. stockleyi* shares with other hyperodapedontines an internal trochanter separated from the femoral head.

### Hyperodapedontine phylogeny

**Figure 11:**
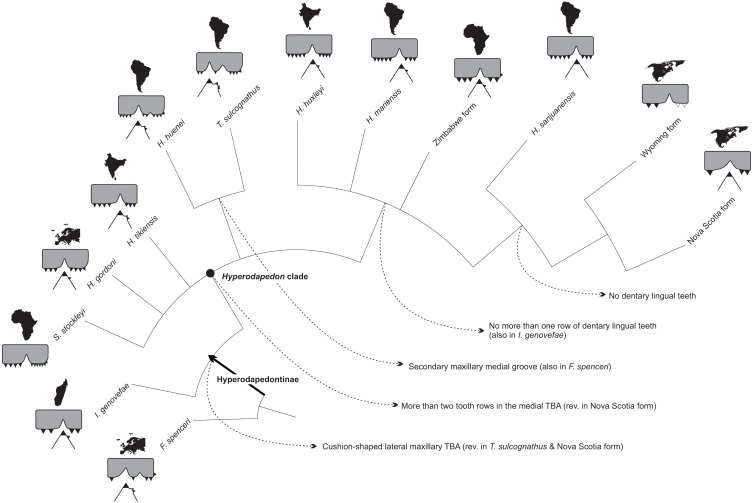
Tooth evolution of Hyperodapedontinae rhynchosaurs, based on the phylogeny of Fig. 10. Each taxon/form represented by a cross section of the maxillo-mandibular apparatus, including the outline of the continent/area it comes from. Dotted lines indicate reconstructed parts. *F. spenceri* represented by EXEMS 60/1985.292; *I. genovefae* by FMNH-9-8-98-525 (from Whatley, 2005); *S. stockleyi* by SAM-PK-11705; *H. gordoni* by NUDG A (from Benton, 1983); *H. tikiensis* by IITKGPR51, (from Mukherjee & Ray, 2014); *H. huenei* by UFRGS PV-0123T; *T. sulcognathus* by UFRGS PV-0298T; *H. huxleyi* by ISIR 17 (from Chatterjee, 1974); *H. mariensis* by UFRGS PV-0408T; Zimbabwe form by BP Z53/3; *H. sanjuanensis* by MCP 1693; Wyoming form by USNM 494329 (from Lucas, Heckert & Hotton III, 2002); Nova Scotia by NSM 012GF032.026 (from Sues & Olsen, 2015).

The phylogenetic relations of hyperodapedontine rhynchosaurs are debated, with slightly different arrangements found in the literature (Langer et al., 2000; Whatley, 2005; Hone & Benton, 2008; Mukherjee & Ray, 2014). The position of *Isalorhynchus genovefae* as the sister-taxon to all other known Hyperodapedontinae is becoming more consensual, as supported in the present study by the distribution of several characters (e.g., 30, 31, 52, 69, 71, 85, 121, 122). As for the *Hyperodapedon*-*Teyumbaita* complex, different features are exclusively (or nearly exclusively) shared by *H. huenei* and *T. sulcognathus*, including a hook-shaped maxillary process at the premaxilla suture, a dorsally grooved frontal, a second crest dorsal to the *anguli oris* crest in the jugal (also seen in *I. genovefae*), a second medial maxillary groove (limited to the caudal region in *H. huenei* and seen in various non-hyperodapedontine rhynchosaurs), a higher number of dentary lingual tooth rows (also seen in *H*. *tikiensis*, some specimens of *H*. *gordoni*, and non-hyperodapedontine rhynchosaurs), and a bulged tooth-bearing area on the medial surface of the caudal portion of the dentary (also seen in some specimens of *H*. *tikiensis*). Accordingly, a sister-group relation between *H. huenei* and *T. sulcognathus* is proposed here (Fig. 9), and would impose nomenclatural changes to prevent the generic epithet *Hyperodapedon* of being applied to a paraphyletic array of taxa. The simplest of the solutions, and perhaps the orthodox one, would be to refer *T. sulcognathus* to the genus *Hyperodapedon*. Yet for the time being, we adopt a Phylogenetic Nomenclature approach (Cantino & De Queiroz, 2010) and simply carry on using *T. sulcognathus* within the “*Hyperodapedon* clade” (Figs. 10 and 11). We chose such an approach because of the remaining uncertain relations within the “*Hyperodapedon* clade”, highlighted by the polytomy at its base (Fig. 10). In addition, we consider that a more comprehensive review of Hyperodapedontinae, including a revaluation of the alpha-taxonomy of the more specimen-rich species of *Hyperodapedon* (*H. gordoni*, *H. huxleyi*, *H. mariensis*, and *H. sanjuanensis*), is needed before nomenclatural changes are applied.

A clade formed by *Hyperodapedon sanjuanensis* and the Nova Scotia (Baird, 1963; Sues & Olsen, 2015) plus Wyoming (Lucas, Heckert & Hotton III, 2002) forms has also been recovered (Fig. 10), matching previous suggestions in the literature (Chatterjee, 1980; Lucas, Heckert & Hotton III, 2002; Mukherjee & Ray, 2014). These taxa share a single tooth row in the dentary (no lingual teeth) and a crest-shaped maxillary medial TBA (uncertain in the Wyoming form). Yet it is important to consider that all specimens attributed to the Nova Scotia and Wyoming rhynchosaurs are reduced in size (possibly representing juveniles). Indeed, dentary lingual teeth may appear latter in ontogeny and a crest-shaped maxillary medial TBA may be, in the Nova Scotia form, constrained by the small number of longitudinal tooth rows in the maxilla (which may also be a factor of ontogeny). However, the record of a *Hyperodapedon* dentary (UFRGS-PV-0413T) with less than 5 cm of estimated length with two rows of lingual teeth (Langer, Ferigolo & Schultz, 2000) compel for taxonomic rather than ontogenetic explanations, suggesting that those North American forms do form a clade with *H. sanjuanensis*. In addition, a reduced number of tooth rows in the rostral part of the maxilla unites the Nova Scotia and Wyoming forms into a clade, a relation that has also to be seen with caution, because the trait may also be related to their early ontogenetic stage. In fact, the scarcity of rhynchosaurs in the Late Triassic of North America is still intriguing, and more complete specimens are needed to fully understand their relation to other Pangean forms.

The remaining relations proposed for Hyperodapedontinae are much more poorly constrained, including the position of *Supradapedon stockleyi*. In fact, the strict consensus (Fig. 10) in the current analysis shows a polytomy including *Su. stockleyi*, *Hyperodapedon gordoni*, *H. tikiensis*, the *H. huenei* plus *Teyumbaita sulcognathus* clade, and another clade including the remaining components of the “*Hyperodapedon* clade”. The latter is supported by the presence of no more than one row of dentary lingual teeth, although *Isalorhynchus genovefae* and some *H. gordoni* and *H. tikiensis* specimens also show this condition (likely as convergences). *H. huxleyi, H. mariensis,* and the Zimbabwe form are found in polytomy within that clade, along with the clade of *Hyperodapedon* lacking lingual teeth (formed by *H. sanjuanensis* and the Wyoming plus Nova Scotia forms). As for *Su. stockleyi*, in none of the 11 recovered MPTs it represents the earliest-branching member of the “*Hyperodapedon*-clade”, so that is assignment to that genus, as proposed by Langer et al. (2000) and Mukherjee & Ray (2014), is possible. Yet following the same reasoning regarding the status of *Teyumbaita* above, we prefer to keep using the generic epithet *Supradapedon* for the Tanzanian hyperodapedontine until detailed taxonomical reviews of other taxa are conducted and a more robust phylogenetic resolution is achieved.

### Hyperodapedontine tooth evolution

Regardless of the poor resolution of the tree, and poor support of some clades, the proposed phylogeny represents an advance over previous hypotheses of Hyperodapedontinae relationships (Benton, 1983; Benton, 1990; Langer et al., 2000; Langer & Schultz, 2000a; Whatley, 2005; Hone & Benton, 2008; Montefeltro, Langer & Schultz, 2010; Montefeltro et al., 2013; Mukherjee & Ray, 2014). It stresses a point previously hinted by authors working with mid Triassic rhynchosaurs (Nesbitt & Whatley, 2004; Langer et al., 2010; Butler et al., 2015; Ezcurra, Montefeltro & Butler, 2015; Schultz, Langer & Montefeltro, 2016), the convergent acquisition of a jaw apparatus composed of two maxillary grooves and dentary blades, instead of a single loss of such arrangement along rhynchosaur evolution. The latter hypothesis was first advocated in a phylogenetic context by Benton (1983) and Benton (1990), suggesting that the plesiomorphic condition for Rhynchosauridae was a set of two mandibular blades and maxillary grooves. This relied on a different understanding of the dental anatomy of some English Middle Triassic forms (Ezcurra, Montefeltro & Butler, 2015), as well as on incomplete knowledge of rhynchosaur diversity (forms such as *Ammorhynchus navajoi*, *Hyperodapedon huenei*, *Brasinorhynchus mariantensis*, *Teyumbaita sulcognathus*, as well as better preserved specimens of *Isalorhynchus genovefae*, were unknown at the time). As such, the only known Late Triassic rhynchosaur at that time with two mandibular blades and maxillary grooves, *T. sulcognathus*, was considered “transitional” between the plesiomorphic Middle Triassic taxa and the other Late Triassic forms (Langer & Schultz, 2000b; Whatley, 2005; Mukherjee & Ray, 2014; Ezcurra, Montefeltro & Butler, 2015). Yet it became clear that various Middle Triassic rhynchosaurs either have an uncertain condition regarding this character (*Bentonyx sidensis*, *Rhynchosaurus articeps*; Langer et al., 2010; Butler et al., 2015; Ezcurra, Montefeltro & Butler, 2015) or actually bear jaws with a single blade/groove (*Ammorhynchus navajoi*, *Langeronyx brodiei*; Nesbitt & Whatley, 2004; Ezcurra, Montefeltro & Butler, 2015). Indeed, more parsimonious reconstructions now show the presence of a single blade/groove jaw apparatus as plesiomorphic for Rhynchosauridae, with the independent acquisition of the double blade/groove apparatus in core stenaulorhynchines (Schultz, Langer & Montefeltro, 2016) and some members of the *Hyperodapedon*-line such as *Fodonyx spenceri* and *T. sulcognathus*.

Another evolutionary pathway previously identified, and highlighted in the present contribution, concerns the reduction of medially placed dental structures (lingual teeth, medial dentary blade, medial maxillary groove, medial TBAs, etc.) in some hyperodapedontines, as epitomized by the condition seen in *Hyperodapedon sanjuanensis* (Azevedo, 1984) and, less clearly because of the scarcity of material, the North American forms (Lucas, Heckert & Hotton III, 2002; Sues & Olsen, 2015). This was previously believed to represent the sole general trend along Hyperodapedontinae tooth evolution (Langer & Schultz, 2000b), starting with a plesiomorphic condition for hyperodapedontines supposedly close to that of *Teyumbaita sulcognathus*, where the medial structures of the jaw are more developed then the lateral ones. Yet the new hypothesis advocated here implies the presence of two opposed evolutionary trends within the group. On one side, *H. sanjuanensis* and the North American forms indeed bear reduced medial dental structures. To a lesser degree, this is seen in the entire clade that also includes *H*. *huxleyi*, *H*. *mariensis*, and the Zimbabwe form (Fig. 11), which is characterized by the presence of no more than a single row of dentary lingual teeth. On the contrary, the opposite trend is seen in the *H. huenei-T*. *sulcognathus* clade, in which features of the medial side of the jaw apparatus are enhanced (Fig. 11). As for the plesiomorphic condition of Hyperodapedontinae, its inference is hampered by the uncertain relations of the terminal taxa on the basal polytomy of the “*Hyperodapedon* clade”. These bear unique sets of plesiomorphic traits and potential synapomorphies of either of the two clades of that polytomy. For example, *Supradapedon stockleyi* has a medial maxillary TBA that is narrower than the lateral, but bears more tooth rows. *H. gordoni* has lateral and medial maxillary TBAs of nearly the same width, but lacks a bulged area on the lingual surface of the dentary. Finally, *H. tikiensis* has more tooth rows on the lateral maxillary TBA, but often more than one lingual tooth row in the dentary. Indeed, future analyses may show that some of these taxa either belong to the two mentioned clades, or have split earlier in hyperodapedontine evolution. More complete additional specimens of rare taxa such as *H. huenei*, *Su. stockleyi*, the Nova Scotia, Zimbabwe, and Wyoming forms, combined with deep revisions of the specimen inclusivity of abundant ones such as *H. huxleyi, H. sanjuanensis*, *H. mariensis*, and *H. gordoni*, as well as data on the biological meaning of such great dental diversity, are needed to reach a more comprehensive understanding of Hyperodapedontinae relationships and tooth evolution.

### Hyperodapedontines as index fossils

The use of *Hyperodapedon* as an index-fossil (Lucas & Heckert, 2002; Langer, 2005) followed its synonimisation with the South American genus *Scaphonyx* (Langer et al., 2000) revealing the broad distribution of the genus in purportedly Carnian stratigraphic units across most of Pangaea. This is enhanced by the supposedly coeval ecological conditions that allowed the genus to represent the most abundant primary consumer in various of those life assemblages (Romer, 1962; Benton, 1983; Langer, 2005). Yet attempts to use tetrapod-based transcontinental correlations to define relative ages to Triassic fossil assemblages (Lucas, 1998; Lucas, 2010) have been heavily criticized (Irmis et al., 2011; Marsicano et al., 2015) including the schemes that segregate the *Hyperodapedon*-rich Late Triassic strata of Pangaea in time; i.e., the Lucas & Heckert (2002) proposal, in which the lower part of the Maleri Formation assemblage, in India, where *H. huxleyi* is found, is considered older than the strata yielding *H. gordoni*, *H. huenei*, *H. mariensis*, and *H. sanjuanensis*.

If the ecological information provided by the abundance of *Hyperodapedon* is of any value, their bearing strata have to be primarily considered of the same age, and then tested by independent evidence. Yet radioisotopic ages for such strata have been restricted to the Ischigualasto Formation, in Argentina (Rogers et al., 1993; Martinez et al., 2011; Martínez et al., 2013), and the Santa Maria Formation, in Brazil (Langer et al., 2017). Both have been dated as late Carnian and are bracketed in time by younger and older deposits of the same basins, which lack the typical *Hyperodapedon*-rich faunas. These correspond to the early Carnian Chañares Formation (Marsicano et al., 2015; Ezcurra et al., 2017) and *Santacruzodon* Assemblage-Zone (Philipp et al., 2013) and the early (although not earliest) Norian Caturrita Formation (Langer et al., 2017) and upper part of the Ischigualasto Formation (Martinez et al., 2011). Indeed, until further information is available, a Carnian to earliest Norian age could be extended to the other Pangaean stratigraphic units where *Hyperodapedon* is highly abundant (i.e., Tiki, lower part of the Maleri, and Lossiemouth Sandstone formations). Also, this generalization can be hinted for units with a less abundant fossil record, but with members of the “*Hyperodapedon* clade” as one of the recognized taxa, such as the Popo Agie and Pebbly Arkose formations, the Evangeline Member of the Wolfville Formation, as well as the here discussed (see above) Mahogo Formation. Indeed, as for the best known North American strata, a recent and comprehensive review of the Fundy Rift Basin (Sues & Olsen, 2015) gathered evidence of an agreeing late Carnian age for the Evangeline Member of the Wolfville Formation, which yielded the Nova Scotia form, and a similar age has also been hinted for the Popo Agie Formation (Hartman, Lovelace & Stocker, 2015), which yielded the Wyoming form. Radiosisotpic dates are still missing for the Tiki, lower part of the Maleri, and Lossiemouth Sandstone formations, but these could test the validity of *Hyperodapedon* as a late Carnian (Tuvalian) index fossil.

As for *Teyumbaita sulcognathus*, its relatively poor fossil record is consistently above that of *Hyperodapedon* spp. in south Brazil (Langer et al., 2007). Indeed, its phylogenetic position as an apical member of the Hyperodapedontinae, rather than as a “transitional” form (see above) better fits its proposed higher stratigraphic provenance. Yet this is admittedly a tentative scenario, to be tested by independent sources of evidence such as the radioisotopic dating of its bearing strata in Brazil. If its stratigraphic/chronologic segregation from the *Hyperodapedon*-rich beds is rejected, *T. sulcognathus* would fit into the general late Carnian outburst of *Hyperodapedon*-like rhynchosaurs. On the contrary, if a younger age is confirmed, we ought to consider *T. sulcognathus* as the single hyperodapedontine to survive into times when the group was already less abundant, until now exclusively in Brazil. Alternatively, given that in a pure phylogenetic understanding, *T. sulcognathus* is no more than an unusual species of *Hyperodapedon*, such a younger record may indicate that the genus is indeed not a strictly Carnian index-fossil, stressing that *Hyperodapedon* species could occur in latter, post-Carnian times.

## Conclusion

The Karoo deposits in the southern margin of the Selous Basin, in southern Tanzania, preserve stratigraphic units that correspond to those of Late Triassic to Early Jurassic age in the central portion of the basin. Considering its lithology, surface distribution in the area, and putative Carnian age, the Mahogo Formation is the most likely source of the fossil remains attributed to the rhynchosaur *Supradapedon stockleyi*. This corresponds to a valid rhynchosaur species within the Hyperodapedontinae clade, which adds to the knowledge of rhynchosaur dental evolution and Late Triassic stratigraphic correlation based on tetrapods.

##  Supplemental Information

10.7717/peerj.4038/supp-1 Supplemental Information 1 Supplementary Infomation and Figures

## Institutional abbreviations

BSPG: Bayerische Staatssammlung für Paläontologie und Geologie, Munich, Germany.
BP: Evolutionary Studies Institute, University of the Witwatersrand, Johannesburg, South Africa.
EXEMS: Royal Albert Memorial Museum, Exeter, UK.
FMNH: Field Museum of Natural History, Chicago, USA.
IGMPT: Institut und Museum für Geologie und Paläontologie, Tübingen, Germany.
IITKGPR: Department of Geology and Geophysics, Indian Institute of Technology, Kharagpur, India.
ISIR: Geological Studies Unit, Indian Statistical Institute, Kolkata, India.
MACN-Pv: Museo Argentino de Ciencias Naturales, Colección Nacional de Paleovertebrados, Buenos Aires, Argentina.
MCP: Museu de Ciências e Tecnologia, Pontifícia Universidade Católica, Porto Alegre, Brazil.
NUGD: Royal Scottish Museum, Edinburgh, UK.
NSM: Nova Scotia Museum of Natural History, Halifax, Nova Scotia, Canada.
PVL: Instituto Miguel Lillo, Universidad Nacional de Tucumán, San Miguel de Tucumán, Argentina.
PVSJ: Museo de Ciencias Naturales, Universidad Nacional de San Juan, San Juan, Argentina.
SAM-PK: Iziko-South African Museum, Cape Town, South Africa;
UFRGS: Instituto de Geociências, Universidade Federal do Rio Grande do Sul, Porto Alegre, Brazil.
USNM: National Museum of Natural History, Smithsonian Institution, Washington D.C., USA.

## Additional Information and Declarations

### Field Study Permissions

The following information was supplied relating to field study approvals (i.e., approving body and any reference numbers): The field work and fossil collection was granted by Excavation/Collector License No: 21/2014/2015 from the Ministry of Natural Resources and Tourism, The United Republic of Tanzania.

### Data Availability

The following information was supplied regarding data availability: The specimens described in the manuscript are stored at the Iziko-South African Museum, Cape Town, South Africa.

## References

[ref-1] Araújo R, Castanhinha R, Junior LC, Lopes FC, Andrade AI, Henriques MH, Quinta-Ferreira M, Reis RP, Barata MT (2012). A new anomodont taxon from the Mozambican Karoo (Niassa Province, Late Permian). Para conhecer a Terra: memórias e notícias de Geociências no espaço lusófono.

[ref-2] Azevedo SAK (1984). Sobre a presença de *Scaphonyx sanjuanensis* Sill, 1970 no Neotriássico do Rio Grande do Sul, Brasil. Pesquisas.

[ref-3] Baird D (1963). Rhynchosaurs in the Late Triassic of Nova Scotia. Geological Society of America, Special Papers.

[ref-4] Benton MJ (1983). The Triassic reptile *Hyperodapedon* from Elgin: functional morphology and relationships. Philosophical Transactions of the Royal Society B: Biological Sciences.

[ref-5] Benton MJ (1984). Tooth form, growth, and function in Triassic rhynchosaurs (Reptilia, Diapsida). Palaeontology.

[ref-6] Benton MJ (1990). The species of *Rhynchosaurus*, a rhynchosaur (Reptilia, Diapsida) from the Middle Triassic of England. Philosophical Transactions of the Royal Society B: Biological Sciences.

[ref-7] Boonstra LD (1953). A note on some rhynchosaurian remains from Tanganyika territory. Annals of the South African Museum.

[ref-8] Butler RJ, Ezcurra MD, Montefeltro FC, Samathi A, Sobral G (2015). A new species of basal rhynchosaur (Diapsida: Archosauromorpha) from the early Middle Triassic of South Africa, and the early evolution of Rhynchosauria. Zoological Journal of the Linnean Society.

[ref-9] Cantino PD, De Queiroz K (2010). http://www.ohio.edu/phylocode.

[ref-10] Catuneanu O, Wopfner H, Eriksson PG, Cairncross B, Rubidge BS, Smith RMH, Hancox PJ (2005). The Karoo basins of south-central Africa. Journal of African Earth Sciences.

[ref-11] Chatterjee S (1969). Rhynchosaurs in time and space. Proceedings of the Geological Society, London.

[ref-12] Chatterjee S (1974). A rhynchosaur from the Upper Triassic Maleri Formation of India. Philosophical Transactions of the Royal Society of London, Series B.

[ref-13] Chatterjee S (1980). The evolution of rhynchosaurs. Mémoires de la Société Géologique de France.

[ref-14] Dilkes D (1998). The early Triassic rhynchosaur *Mesosuchus browni* and the interrelationships of basal archosauromorph reptiles. Philosophical Transactions of the Royal Society of London B: Biological Sciences.

[ref-15] Ezcurra MD, Fiorelli LE, Martinelli AG, Rocher SM, Von Baczko B, Ezpeleta M, Taborda JRA, Hechenleitner EM, Trotteyn MJ, Desojo JB (2017). Deep faunistic changes preceded the raise of dinosaurs 1 in southwestern Pangaea. Nature Ecology & Evolution.

[ref-16] Ezcurra MD, Montefeltro FC, Butler RJ (2015). The early evolution of rhynchosaurs. Frontiers in Ecology and Evolution.

[ref-17] Goloboff P, Farris J, Nixon K (2008). TNT, a free program for phylogenetic analysis. Cladistics.

[ref-18] Hankel O (1987). Lithostratigraphic subdivision on the Karoo rocks of the Luwegu Basin (Tanzania) and their biostratigraphic classification based on microfloras, macrofloras, fossil wood and vertebrates. Geologische Rundschau.

[ref-19] Hartman S, Lovelace DM, Stocker ML (2015). Stratigraphic and chronologic relationships of the Popo Agie Formation, Upper Chugwater Group.

[ref-20] Hone DWE, Benton MJ (2008). A new genus of rhynchosaur from the Mid Triassic of SW England. Palaeontology.

[ref-21] Horn BLD, Schultz CL, Figueiredo AEQ, Motta FA (2015). Recognition of the *Hyperodapedon* assemblage zone (Late Triassic) in a relictual occurrence over the Sul-Rio-Grandense Shield. Revista Brasileira de Paleontologia.

[ref-22] Hunt AP, Lucas SG (1991). A new Rhynchosaur from the Upper Triassic of West Texas, and the Biochronology of Late Triassic Rhyncosaurs. Palaeontology.

[ref-23] Irmis RB, Mundil R, Martz JW, Parker WG (2011). High-resolution U-Pb ages from the Upper Triassic Chinle Formation (New Mexico, USA) support a diachronous rise of dinosaurs. Earth and Planetary Science Letters.

[ref-24] King BC, Ramberg IB, Neumann ER (1978). A comparison between the older (Karroo) rifts and the younger (Cenozoic) rifts of eastern Africa. Tectonics and geophysics of continental rifts.

[ref-25] Kreuser T, Wopfner H, Kaaya CZ, Markwort S, Semkiwa PM, Aslanidis P (1990). Depositional evolution of Permo-Triassic Karoo basins in Tanzania with reference to their economic potential. Journal of African Earth Sciences.

[ref-26] Langer MC (2005). Studies on continental Late Triassic tetrapod biochronology. II. The Ischigualastian and a Carnian global correlation. Journal of South American Earth Sciences.

[ref-27] Langer MC, Boniface M, Cuny G, Barbieri L (2000). The phylogenetic position of *Isalorhynchus genovefae*, a Late Triassic rhynchosaur from Madagascar. Annales de Paléontologie.

[ref-28] Langer MC, Ferigolo J, Schultz CL (2000). Heterochrony and tooth evolution in hyperodapedontine rhynchosaurs (Reptilia, Diapsida). Lethaia.

[ref-29] Langer MC, Montefeltro FC, Hone DWE, Whatley R, Schultz CL (2010). On *Fodonyxspenceri* and a new rhynchosaur from the Middle Triassic of Devon. Jounal of Vertebrate Paleontology.

[ref-30] Langer MC, Ramezani J, Dias-da-Silva S, Cabreira S, Pretto F, Bronzati M, Marsola J, Müller R, Pacheco C, Roberto-da-Silva L (2017). New dinosauromorphs and radioisotopic ages from the Late Triassic Santa Maria and Caturrita formations, south Brazil. Journal of Vertebrate Paleontology Program and Abstracts.

[ref-31] Langer MC, Ribeiro AM, Schultz CL, Ferigolo J (2007). The continental tetrapod–bearing Triassic of South Brazil. Bulletin of the New Mexico Museum of Natural History and Science.

[ref-32] Langer MC, Schultz CL (2000a). A new species of the late Triassic rhynchosaur *Hyperodapedon* from the Santa Maria Formation of south Brazil. Palaeontology.

[ref-33] Langer MC, Schultz CL, Holz, Roz LF (2000b). Rincossauros- herbívoros cosmopolitas do Triássico [Rincosauros- cosmopolitan herbivores of the Triassic]. Paleontologia do Rio Grande do Sul.

[ref-34] Lucas SG (1998). Global Triassic tetrapod biostratigraphy and biochronology. Palaeogeography, Palaeoclimatology, Palaeoecology.

[ref-35] Lucas SG (2010). The Triassic timescale based on nonmarine tetrapod biostratigraphy and biochronology. Geological Society Special Publication.

[ref-36] Lucas SG, Heckert AB (2002). The *Hyperodapedon* Biochron, Late Triassic of Pangea. Albertiana.

[ref-37] Lucas SG, Heckert AB, Hotton III N (2002). The rhynchosaur *Hyperodapedon* from the upper Triassic of Wyoming and its global biochronological significance. Bulletin of the New Mexico Museum of Natural History and Sciences.

[ref-38] Marsicano CA, Irmis RB, Mancuso AC, Mundil R, Chemale F (2015). The precise temporal calibration of dinosaur origins. Proceedings of the National Academy of Science of the United States of America.

[ref-39] Martínez RN, Apaldetti C, Alcober OA, Colombi CE, Sereno PC, Fernandez E, Malnis PS, Correa GA, Abelin D (2013). Vertebrate succession in the Ischigualasto Formation. Journal of Vertebrate Paleontology.

[ref-40] Martinez RN, Sereno PC, Alcober OA, Colombi CE, Renne PR, Montañez IP, Currie BS (2011). A basal dinosaur from the dawn of the dinosaur era in southwestern Pangaea. Science.

[ref-41] Miller RM, Selley RC (1997). The Ovambo basin of northern Namibia. African Basins, Sedimentary Basins of the World, 3.

[ref-42] Montefeltro FC, Bittencourt JS, Langer MC, Schultz CL (2013). Postcranial anatomy of the hyperodapedontine rhynchosaur *Teyumbaita sulcognathus* (Azevedo and Schultz, 1987) from the Late Triassic of southern Brazil. Journal of Vertebrate Paleontology.

[ref-43] Montefeltro FC, Langer MC, Schultz CL (2010). Cranial anatomy of a new genus of hyperodapedontine rhynchosaur (Diapsida, Archosauromorpha) from the upper Triassic of southern Brazil. Earth and Environmental Science Transactions of the Royal Society of Edinburgh.

[ref-44] Mukherjee D, Ray S (2014). A new *Hyperodapedon* (Archosauromorpha, Rhynchosauria) from the upper Triassic of India: implications for rhynchosaur phylogeny. Palaeontology.

[ref-45] Nesbitt SJ, Butler RJ, Ezcurra MD, Barrett PM, Stocker MR, Angielczyk KD, Smith RMH, Sidor CA, Niedźwiedzki G, Sennikov AG, Charig AJ (2017). The earliest bird-line archosaurs and the assembly of the dinosaur body plan. Nature.

[ref-46] Nesbitt SJ, Whatley RL (2004). The first discovery of a rhynchosaur from the upper Moenkopi Formation (Middle Triassic) of northern Arizona. PalaeoBios.

[ref-47] Philipp RP, Closs H, Schultz CL, Basei MB, Horn LD, Soares MB (2013). Proveniência por U-Pb LA-ICP-MS em zircão detrítico e idade de deposição da Formação Santa Maria, Triássico da Bacia do Paraná, RS: evidências da estruturação do Arco do Rio Grande.

[ref-48] Piqué A, Laville E, Bignot G, Rabarimanana M, Thouin C (1999). Louverture et le développement du bassin de Morondava (Madagascar) du Carbonifère supérieur au Jurassique moyen. Données stratigraphiques, sédimentaires, paléontologiques et structurales [Louverture and the development of Upper Carboniferous Morondava Basin (Madagascar) in the Middle Jurassic. Stratigraphic, sedimentary, paleontological data and structural]. Journal of African Earth Sciences.

[ref-49] Rogers RR, Swisher III CC, Sereno PC, Monetta AM, Forster CA, Martinez RN (1993). The ischigualasto tetrapod assemblage (Late Triassic, Argentina) and 40Ar/39Ar dating of dinosaur origins. Science.

[ref-50] Romer AS (1962). La evolución explosiva de los rhynchosaurios del Triásico [The explosive evolution of the rhynchosaurs of the Triassic]. Revista del Museo Argentino de Ciencias Naturales Bernardino Rivadavia e Instituto Nacional de Invetigacion de las Ciencias Naturales. Ciencias Zoológicas.

[ref-51] Rosendahl BR (1987). Architecture of continental rifts with specialreference to East Africa. Annual Review of Earth and Planetary Sciences.

[ref-52] Schultz CL, Langer MC, Montefeltro FC (2016). A new rhynchosaur from south Brazil (Santa Maria Formation) and rhynchosaur diversity patterns across the Middle-Late Triassic boundary. Paläontologische Zeitschrift.

[ref-53] Stockley GM (1931). Report on the geology of the Ruhuhu coalfields. Geological Survey of Tanganyika, Bulletin.

[ref-54] Stockley GM (1936). A further contribution on the karroo rocks of tanganyika territory. Quarterly Journal of the Geological Society of London.

[ref-55] Stockley GM (1943). The geology of the rufiji district including a small portion of the north Kilwa District (Matumbi Hills). Tanganyika Notes and Records.

[ref-56] Stockley GM (1946). Phosphate deposits in Tanganyika Territory, with special reference to the Zizi Apatite-limestone, South of Kisaki. The East African Agricultural Journal.

[ref-57] Stockley GM (1947a). New coal discoveries in Tanganyika. Mineralogy Magazine.

[ref-58] Stockley GM (1947b). The geology and mineral resources of Tanganyka Territory. Bulletin of the Imperial Institute.

[ref-59] Sues H-D, Olsen PE (2015). Stratigraphic and temporal context and faunal diversity of Permian-Jurassic continental tetrapod assemblages from the Fundy rift basin, eastern Canada. Atlantic Geology.

[ref-60] Thornton W (2014). Western Tanzania: the Lake Rukwa and Lake Nyasa rifts. Geoexpro.

[ref-61] Verniers J, Jourdan P, Paulis R, Frasca-Spada L, De Bock F (1989). The Karroo graben of Metangula, Northern Mozambique. Journal African Earth Sciences.

[ref-62] Whatley R (2005). Phylogenetic relationship of Isalorhynchus genovefae, the Rhynchosaur (Reptilia, Archosauromorpha) from Madagascar. PhD dissertation.

[ref-63] Wopfner H (1994). The Malagasy Rift, a chasm in the Tethyan margin of Gondwana. Journal of Southeast Asian Earth Sciences.

[ref-64] Wopfner H (2002). Tectonic and climatic events controlling deposition in Tanzanian Karoo basins. Journal of African Earth Sciences.

