# Supplementary material for: Supradapedon revisited: geological explorations in the Triassic of southern Tanzania
Source: PeerJ. 2017 Nov 13;5:e4038. doi: 10.7717/peerj.4038 (PMC5689022; doi:10.7717/peerj.4038)
Supplement: Supplemental Information 1 [file peerj-05-4038-s001.pdf]

Supplementary Material to:

Supradapedon revisited: geological explorations in the Triassic of southern Tanzania

Max C. Langer [1], Átila A. S. da Rosa [2], Felipe C. Montefeltro [3]

[1] Laboratório de Palentologia, FFCLRP, Universidade de São Paulo, Ribeirão Preto, SP, Brazil

[2] Laboratório de Estratigrafia e Paleobiologia, Universidade Federal de Santa Maria, Santa Maria, RS, Brazil

[3] Departamento de Biologia e Zootecnia, FEIS, Universidade Estadual Paulista, Ilha Solteira, SP, Brazil

This document includes:

1 - CHARACTER LIST

2 - OTUs USED IN THE PHYLOGENETIC ANALYSIS

3 – TAXON-CHARACTER MATRIX

4 – SYNAPOMORPHY LIST

5 – BOOTSTRAP AND BREMER-SUPORT VALUES

1 – CHARACTER LIST

Character list used in the parsimony analysis. Characters modified from previously published analyses, and new characters are acknowledged accordingly. Characters 70 and 76 are treated as additive.

1. Skull dimensions: longer than broad (0); broader than long (1) (Benton, 1984).
2. Skull height: <50% of the midline length (0); >50% of the midline length (1) (Hone & Benton, 2008).
3. External nares: separate (0), single medial naris (1) (Benton, 1985).
4. Orbit orientation: mostly lateral (0); mostly dorsal (1) (Langer & Schultz, 2000).
5. Orbit with elevated rim along the jugal, postorbital, frontal, prefrontal and lacrimal: absent (0); present (1).
6. Orbital medial margin: rounded (0); forming a marked angle (1) (Montefeltro, Langer & Schultz, 2010).
7. Lower temporal fenestra: open ventrally (0), closed ventrally (1) (Dilkes, 1998).
8. Premaxilla ventral margin: horizontal (0), down-turned (1) (Benton, 1985).
9. Premaxilla and prefrontal contact: absent (0), present (1) (Dilkes, 1998).
10. Shape of rostral margin of nasal at midline: strongly convex with rostral process (0) or transverse with little convexity (1) (Dilkes, 1998).
11. Jugal and maxillary heights bellow the ventral border of the orbit: maxilla higher (0); jugal higher (1) (Benton, 1984).
12. Jugal-lacrimal contact: minimal (0); extensive contact of the jugal rostral process (1) (Whatley, 2005).
13. Anguli oris crest: absent (0); present (1) (modified from Benton, 1984).
14. Rostral extension of the anguli oris crest: restricted to the main body of the jugal (0); extending onto the rostral process of the jugal, but not the maxilla (1); extending onto the maxilla, but not the rostral process of the jugal (2) (modified from Benton, 1984).
15. Jugal surface dorsal to anguli oris crest: lacking a secondary crest (0); with a secondary anguli oris crest (1) (Langer & Schultz, 2000).
16. Lateral overlap of maxilla by jugal: absent or minimally expanded (0); well developed (1) (Whatley, 2005).

17. Jugal with multiple pits on the lateral surface of its main body: absent (0); present (1) (Butler et a. 2015).
18. Jugal subtemporal process: height >50% of the length (0); height <50% of the length (1) (Dilkes, 1995).
19. Jugal subtemporal process with a distinct lateroventral orientation with respect to the sagittal axis of the snout: absent (0); present (1) (Butler et al. 2015).
20. Relative widths of postorbital bar and lower temporal fenestra: <0.4 (0); >0.4 (1) (Langer & Schultz, 2000).
21. Dorsomedial surface of prefrontal near the orbital rim: flat or slightly concave (0); deeply concave (1) (Whatley, 2005).
22. Procumbent lacrimal and prefrontal rostrolateral margin: absent (0); present (1) (Whatley, 2005).
23. Groove on the dorsal surface of the frontal: absent (0); present (1) (Dilkes, 1995).
24. Dorsal groove on frontal: longitudinally extended along most of the surface of the frontal (0); rostrolaterally-to-caudomedially extended along the caudal half of the frontal (1) (Butler et al. 2015).
25. Well-marked 'V'-shaped crest along frontal-postfrontal contact, rostral to the margin of the supratemporal fossa: absent (0); present (1) (Montefeltro, Langer & Schultz, 2010).
26. Frontal and parietal midline lengths: frontal longer (0); parietal longer (1) (Benton, 1987).
27. Postfrontal: excluded from upper temporal fenestra border (0); forming the upper temporal fenestra border (1) (Dilkes, 1998).
28. Postfrontal dorsal surface: flat (0); markedly concave (1) (Dilkes, 1995).
29. Caudal extension of the caudal process of the postorbital: considerably rostral to the level of the caudal border of the infratemporal fenestra (0); at level with the caudal border of the infratemporal fenestra (1) (modified from Dilkes, 1998).
30. Postorbital rostroventral process: expanding ventral to the level of the orbital midpoint (0); expanding dorsally to orbital height midpoint (1) (Dilkes, 1998).
31. Postorbital ventral process: expands rostral to the jugal (0); fits dorsal to the jugal (1) (Whatley, 2005).
32. Postorbital-parietal suture: visible in dorsal view (0); hidden in dorsal view (1) (Dilkes, 1998).
33. Parietals: separate (0), fused (1) (Benton, 1985).

34. Parietal foramen: always, or sometimes, present (0), always absent (1) (Benton, 1985).
35. Parietal table: broad (0), constricted and with sagittal crest (1) (Dilkes, 1998).
36. Parietal body: not expanded laterally at midlength (0); expanded laterally at midlength (1) (Montefeltro, Langer & Schultz, 2010).
37. Parietal transverse process: caudolaterally directed (0); laterally directed (1) (Montefeltro, Langer & Schultz, 2010).
38. Distal tip of parietal transverse process: not rostroventrally curved (0); rostroventrally curved (1) (modified from Montefeltro, Langer & Schultz, 2010).
39. Squamosal ventral process: thinner than 50% of dorsoventral length (0); broader than over 50% of dorsoventral length (1) (Benton, 1990).
40. Squamosal medial process: short, forming less than half of the caudal border of the supratemporal fenestra (0); long, forming entire or almost entire caudal border of the supratemporal fenestra (1) (Butler et al. 2015).
41. Relative position of quadratojugal and squamosal processes: squamosal ventral process rostral to quadratojugal dorsal process (0); squamosal ventral process overlapping the quadratojugal dorsal process (1) (Whatley, 2005).
42. Quadratojugal rostral process: completely absent (0), present (1) (Dilkes, 1998).
43. Supratemporal: present (0); absent (1) (Benton, 1984).
44. Supratemporal with a bifurcated medial border, in which a ventromedial process extends underneath the caudolateral process of the parietal: present (0); absent (1) (Butler et al. 2015).
45. Pterygoid midline suture length: greater than or equal to the distance between the caudal margin of the suture and the basipterygoid articulation (0); less than the distance between the caudal margin of the suture and the basipterygoid articulation (1) (Whatley, 2005).
46. Ectopterygoid reaches lateral corner of transverse flange of pterygoid: no (0), yes (1). (Dilkes, 1998)
47. Elements forming the border of the suborbital fenestra: pterygoid, ectopterygoid, palatine, and maxilla (0); pterygoid, ectopterygoid and palatine only (1) (Dilkes, 1995).
48. Supraoccipital shape: plate-like (0); inverted V-shape (1). (Dilkes, 1995)
49. Occipital condyle position: rostral to craniomandibular articulation (0); aligned to craniomandibular articulation (1) (Benton, 1984).

50. Basioccipital and basisphenoid/parasphenoid lengths: basisphenoid/parasphenoid longer (0); basioccipital longer (1) (Langer & Schultz, 2000).
51. Relative positions of the basipterygoid process of the basisphenoid and the ectopterygoid process of the pterygoid: at the same level (0), basipterygoid process of the basisphenoid caudal to ectopterygoid process of the pterygoid (1) (Dilkes, 1995).
52. Basipterygoid process dimensions (dorsoventral length, rostrocaudal width): longer than wide (0); wider than long (1) (Langer & Schultz, 2000).
53. Jaw symphysis: formed largely by dentary (0), formed only by splenial (1) (Dilkes, 1998).
54. Divergence of dentaries in front of symphysis: absent (0), present (1) (Dilkes, 1998).
55. Mandible depth: <0.25 of the total length (0); >0.25 of the total length (1) (Benton, 1984).
56. Dentary length: half, or less, than the total mandibular length (0); greater than half of the total mandibular length (1) (Benton, 1990).
57. Premaxillary teeth: present (0), absent (1) (Benton, 1985).
58. Tooth implantation: subthecodont or thecodont (0), ankylothecodont (1) (Benton, 1985).
59. Maxilla occlusal ventral margin: horizontal (0), convex (1). (modified from Dilkes, 1998).
60. Tooth occlusion: single sided overlap (0), flat occlusion (1), blade and groove jaw apparatus, where dentary blade(s) fit precisely into maxillary groove(s) (2) (Benton, 1985).
61. Vomerine teeth: present (0), absent (1) (Dilkes, 1998).
62. Palatine teeth: present (0), absent (1) (Dilkes, 1998).
63. Pterygoid teeth: present (0), absent (1). (Modified from Benton, 1983a and Ezcurra et al. 2015).
64. Pterygoid teeth: present as two or more rows of bottom-like teeth (0); present, but single distinct row of teeth (1) (Benton, 1983a, 1983a and Ezcurra et al. 2015).
65. Medial maxillary groove: absent (0); present (1) (Benton, 1984).
66. Medial maxillary groove: not reaching the rostral half of the maxilla (0), reaching the rostral half of the maxilla (1) (Benton, 1984).

67. Maxillary area lateral to main groove: narrower than the medial area (0); same width or broader than the medial area (1) (Benton, 1990).
68. Maxillary cross-section lateral to main groove: crest-shaped (0); cushion-shaped (1) (Langer, Ferigolo & Schultz, 2000).
69. Tooth rows lateral to main maxillary groove: a single clear row (0); more than one clear row (1) (Langer & Schultz, 2000).
70. Number of tooth rows medial to main maxillary groove (at the caudal half of the maxilla): single row (0), two rows (1); three or more tooth rows (2) (Langer, Ferigolo & Schultz, 2000, modified from Schultz et al. 2016).
71. Occlusal tooth rows on the rostral half of the maxilla: four or more tooth rows (0); fewer than four tooth rows (1) (Whatley, 2005).

    Obs.: this character surely has some degree of overlap with the previous one, as taxa with fewer tooth rows medial to the main maxillary groove at the caudal half of the maxilla (states “0” and “1” of char. 70) will tend also to have fewer tooth rows on the rostral half of the maxilla (state “1” of char. 71). Yet, taxa with a single (state “0” of char. 70) or even two (state “1” of char. 70) tooth rows medial to the main maxillary groove may also have four or more tooth rows on the rostral half of the maxilla (state “0” of char. 71), because of a higher number of tooth rows lateral to the main maxillary groove. On the contrary, *Brasinorhynchus mariantensis* (Schultz et al., 2016), has three tooth rows (state “2” of char. 70) medial to the main maxillary groove (at the caudal half of the maxilla), but less than four tooth rows on the rostral half of the maxilla (state “1” of char. 71), because not all tooth row on the caudal half of the maxilla reach the rostral part of the bone. Hence, characters 70 and 71 are both biologically and logically independent, not adding strictly duplicate information to the analysis.
72. Maxillary lingual teeth: absent (0); present (1) (Benton, 1984).
73. Maxillary lingual teeth: scattered teeth (0); large number of teeth on the medial surface of the bone (1) (Benton, 1984).
74. Maxillary teeth: only conicals (0); conicals and ‘pyramidal’ (1) (Whatley, 2005).
75. Dentary teeth: only conical (0); conical and rostrocaudally compressed (1) (Whatley, 2005).
76. Number of rows of teeth on dentary: one (0), two (1), more than two full rows (2). (Benton, 1983a).
77. Caudal-most dentary teeth: on the rostral half of lower jaw (0); on the caudal half of lower jaw (1) (Langer & Schultz, 2000).
78. Lingual dentary teeth: absent (0); present (1) (Benton, 1984).

79. Lingual dentary teeth: forming one row (0); forming more than one row (1) (Benton, 1984).
80. Dentary teeth on the dentary lingual surface: forming rows of well-spaced teeth (0); crowded (1) (Benton, 1985, rewritten from Schultz et al. 2016).
81. Axis ventral keel: present (0); absent (1) (Montefeltro *et al*., 2013).
82. Axial parapophysis: present (0); absent (1) (Montefeltro *et al*., 2013).
83. Cervical postaxial vertebrae ventral keel: absent (0); present (1) (Montefeltro *et al*., 2013).
84. Truncal vertebrae with ossified intercentrum: present (0); absent (1) (Evans, 1988).
85. Epipophyses on cervical postzygapophyses: spine-shaped (0); crest-shaped (1) (Whatley, 2005).
86. Position of the transverse process of cranial truncal vertebrae: at the level of prezygapophysis (0); caudally located in the vertebra centrum (1) (Montefeltro *et al*., 2013).
87. Second sacral vertebra: with a notch between the iliac articular surface and the caudal process (0); caudal process continuous to the iliac articular surface (1) (Dilkes, 1998).
88. Caudal vertebrae, neural spine height versus proximodistal length at its base in proximal caudal vertebrae: <2.20 (0); ≥2.20 (1) (Dilkes, 1998).
89. Interclavicle: caudal process longer than twice the lateral processes (0); caudal process shorter than twice the lateral process (1) (Dilkes, 1998).
90. Supinator process on the external surface of humeral ectepicondyle: hook-shaped (0); formed by a low supinator ridge and ligament groove (1) (Montefeltro *et al*., 2013).
91. Caudal process of the coracoid: present (0); absent (1) (Benton, 1984).
92. Dorsal margin of the ilium: cranial process <15% of the length of the caudal process (0); cranial process >15% of the length of the caudal process (1) (Dilkes, 1995).
93. Pubic tubercle on the lateral surface of the pubic shaft: present (0); absent (1) (Whatley, 2005).
94. Internal trochanter: continuous with the femoral head (0); separated from femoral head (1) (Whatley, 2005).
95. Crest on craniomedial region of tibial shaft: absent (0); present (1) (Montefeltro *et al*., 2013).

96. Relative size of astragalar articular facets: tibial facet greater than centrale facet (0); centrale facet greater than tibial facet (1) (Langer & Schultz, 2000).
97. Metatarsal I: longer than broad (0); broader than long (1) (Hone & Benton, 2008).
98. Sacral vertebrae and ribs, shape of the caudal projection of the bifurcated distal end of the second sacral rib: tapering (0); squared (1) (Dilkes 1998).
99. Caudal vertebrae, median longitudinal groove on the ventral surface of the centrum of the first two caudals: absent (0); present (1) (Dilkes, 1998).
100. Metatarsus, length of metatarsal I versus metatarsal III: <0.45 (0); ≥ 0.45 (1) (Dilkes 1998).
101. Caudal orbital margin and caudal margin of maxillary tooth bearing area: at the same level (0), caudal orbital margin located caudally (1) (Schultz *et al.* 2016).
102. Dorsal nasal profile in lateral view: nasal inclined rostrally (0); nasal horizontal oriented, rostral edge in level with caudal edge (1) (Schultz *et al.* 2016).
103. Participation of the frontal in orbital margin: frontal forming great part of the median section of orbital dorsal border (0), prefrontal and postfrontal closer to, or contacting each other reducing the participation of the frontal in dorsal orbital border (1) (Schultz *et al.* 2016).
104. Squamosal body: restricted, forming a restricted intertemporal bar (0), developed and forming a plate-like intertemporal bar (1) (Schultz *et al.* 2016).
105. Rounded depression on the ventral surface of basisphenoid: absent (0), present (1) (Schultz *et al.* 2016).
106. Dentary medial surface at caudal portion: flat (0), forming a bulged area projecting medially from the remaining area of the dentary (1) (Schultz *et al.* 2016).
107. Curvature of the maxillary caudal border in lateral view: caudal border of maxillae curves gentle towards the jugal (0), caudal border of maxillae curves strongly towards the jugal so that the caudal teeth face caudoventrally (1) (Schultz *et al.* 2016).
108. Extension of the lateral, or only, maxillary groove: along the whole occlusal surface of maxilla (0); limited to the caudal half of maxillary occlusal surface (1) (Schultz *et al.* 2016).
109. Maxillary cross-section medial to main groove: crest-shaped (0); cushion-shaped (1) (Schultz *et al.* 2016).

110. Size of maxillary occlusal teeth: enlarged teeth with each longitudinal row formed by reduced number of teeth (0), reduced teeth with each longitudinal row formed by great number of teeth (1) (Schultz *et al.* 2016).
111. Maxillary lingual teeth: restricted to caudal half of the maxilla (0), extending to the rostral half of maxillary length (1) (Schultz *et al.* 2016).
112. Maxillary lingual tooth crown orientation: medially oriented, perpendicular to maxillary medial wall (0), ventrally directed parallel to maxillary medial wall (1) (Schultz *et al.* 2016).
113. Jugal height/length ratio: < 0.5 (0), >0.5 (1) (Modified from Mukherjee & Ray 2014: 12).
114. Frontal pair: Longer than broad (0), Broader than long (1) (Benton 1983, Mukherjee & Ray 2014: 14).
115. Proportion of maxillary tooth plate: Width at caudal end/Length < 0.3 (0), >0.3 (1) (Modified from Mukherjee & Ray 2014: 33).
116. Three bulbous processes or convexities on the cranial surface of the axial centrum: absent (0), present (1) (Mukherjee & Ray 2014: 50).
117. Coracoid foramen: restricted to coracoid (0), shared between scapula and coracoid (1) (Modified from Langer & Schultz 2000, Mukherjee & Ray 2014: 58).
118. Ratio between humeral (HL) and femoral (FL) lengths: HL/FL < 1 (0), HL/FL ≥ 1 (1) (Mukherjee & Ray 2014: 65).
119. Presence of a hook-shaped process at the maxilla-premaxilla suture above the dentigerous area: absent (0), present (1) (new character).
120. Paraoccipital process of opisthotic: curved ventrally (0), straight (1) (new character).
121. Number of tooth rows lateral to the main groove: lesser than the number of medial tooth row (0), as many as or greater than the number of medial tooth rows (1) (new character).
122. Size of teeth in rows L1 and L2: same size as other teeth (0), teeth in L1 or L2 larger than other teeth (1) (new character).
123. Medial most row of occlusal tooth at caudal region of maxilla: strictly occlusal (0), medially displaced and crowns with not strictly occlusal direction (1) (new character).

2 - OTUs USED IN THE PHYLOGENETIC ANALYSIS

Ingroup and outgroup operational taxonomic units used in the phylogenetic analysis. Sources of data for scoring are listed for each taxon (specimens studied firsthand and published descriptive accounts).

Ammorhynchus navajoi — Nesbitt & Whatley (2004).

Bentonyx sidensis — BRSUG 27200; Hone and Benton (2008); Langer et al. (2010).

Eohyosaurus wolvaardti — SAM-PK-K10159; Butler et al. (2015).

Fodonyx spenceri — EXEMS 60/1985.292; Benton (1990).

Brasinorhynchus mariantensis— UFRGS-PV-0168T, UFRGSPV- 0315T.

Howesia browni — SAM-PK-K5884, SAM-PKK5885, SAM-PK-K5886; Dilkes (1995).

Hyperodapedon gordoni — NHMUK R699, NHMUK G281; Benton (1983).

Hyperodapedon huenei — UFRGS-PV-0132T; Langer & Schultz (2000).

Hyperodapedon huxleyi — Chatterjee (1974).

Hyperodapedon mariensis — FZB-PV-1867, FZB-PV-3509, UFRGS-PV-0149T, UFRGS-PV-0262T, UFRGS-PV-0408T, UFRGS-PV-0504T.

Hyperodapedon sanjuanensis — MACN 18185, PVSJ 574, BSG-18.4, BSG-19.2, BSG-19.4, BSG-23A, MCP-1693.

Hyperodapedon tickensis — Mukherjee & Rey (2014).

Hyperodapedon sp. Nova Scotia — Baird (1963), Sues & Olsen (2015).

Hyperodapedon sp. Zimbabwe — Raath et al. (1992).

Hyperodapedon sp. Wyoming — Lucas et al. (2002).

Isalorhynchus genovefae — MDE-R18, MDE-R20; Buffetaut (1983); Whatley (2005).

Langeronyx brodiei — WARMS G950, WARMS G955, WARMS G959, WARMS G960, WARMS G4715, WARMS G6097-1, NHMUK R8495; Benton (1990).

Mesodapedon kuttyi — Chatterjee (1980).

Mesosuchus browni — SAM-PK-K5882, SAMPK- K6536, SAM-PK-K7416, SAM PK-K7701; Dilkes (1998).

Prolacerta broomi — Modesto & Sues (2004).

Protorosaurus speneri — Gottman-Quesada & P. M. Sander (2009).

Rhynchosaurus articeps — SHYMS G-132/1982, SHYMS G-134/1982, SHYMS 3, SHYMS G-133/1982, SHYMS 5, SHYMS 6, SHYMS 7, NHMUK R1236; Benton (1990).

Stenaulorhynchus stockleyi —GPTI/RE/7192; Huene (1938).

Teyumbaita sulcognathus — UFRGS-PV-0232T, UFRGS-PV-0298T, UFRGS-PV-0290T; Montefeltro et al. (2010, 2013).

3 – TAXON-CHARACTER MATRIX

Taxon-character matrix used in the phylogenetic analysis. Missing data are marked as ‘?’, nonapplicable characters as ‘-’, and variable condition within ‘[ ]’.

Ammorhynchus navajoi
???1112????0-
00011110???11??????????????????????????100111??0?????00?

Bentonyx sidensis
001011?1111110000?10101101011?01111110?1?1011?11011011??1112111-
???001?10??2?110???????????????????110110100011?0????010?0

Brasinorhynchus mariantensis 0110000111101200011010 0-
1101100111101 10?010???01010011??1112110111000211100 2?111110??????????????
???10111111011?1000??0100?

Eohyosaurus wolvaardti
00?00?0???0?12001100??????0?100?1?1000000100???0???0??00?1110?00------
?10002?110????????????????????1??0?00--0?01??????0???

Fodonyx spenceri
11?01?0???1?1000?11??????????0???1????0?0????11?????11011112111-
10000111000201 10???????????????????????1?110100?????????000

Hyperodapedon gordoni 11110011111111010111?1100100111011111011111-
11111101111111112111-0-011[12]00-11111001111 10?0011111111??00101001010--
10111100100

Hyperodapedon huenei 11110011111111110111110-0110111111111011111-
01111101111111112111-
100112010112111 0???????????????????010101101000101??11101

Hyperodapedon huxleyi 11110011111111010011?1100110111111111011111-
11111101111111112111-0-211200-111110000?110101111?1111??00101001000--
10101100111

Hyperodapedon mariensis 111100111111110100111110011011111111111011111-
[01][01]11110111111112111-0-211200-1111100???1?00?011??1?1?1??0101001010--
101?100010[01]

Hyperodapedon sanjuanensis 111100111111101011111100110111111111011111-
01111101111111112111-0-211200-11010--?011100?0111?111?1??0101001000--
1010??00100

Hyperodapedon tikiensis
??11???11?1?110?0??????????????????????????????????11?01???1112?11-0-211200-
112?1100111?0???1?1?111????????0?10?0--??11????101

Hyperodapedon Nova Scotia
???????1???1?1????11?2??1-0-
[12]0001??110?0--???????????????????????????00?0?0????0?????100

Hyperodapedon Zimbabwe
??1????1??1?1??1??????????????????????????????1???????1??1112?11-0-111[12]00-
11[12]?1?0??????????????????????????1010--??1???0?100

Hyperodapedon Wyoming
???????1???11?2????0-?01110-
110?0--??????????????????????????????0?0--??0?????11?

Howesia browni 00?0000??10??---
1100001000010?00111000?0??0?10000000?????111?000------
0100020???0??0?001???00000010???0?0?0--01??00?????--1

Isalorhynchus genovefae
11110?1111111111011101?????0100?111?1?1?11??0?1?110011111112111-0-
[12][01]0[12]10-1111100??11000011110111?1?00???001010--1?1??00?010

Langeronyx brodiei ??1010?11?111[02]000?1?100-
0??0?00????????????????0????011??1112????0-
000[01]110002?110?????????????????????000??01-000??0??????010

Mesodapedon kuttyi
??1???1?2????110011111002?
111??????????????????????????????011???0?????00?

Mesosuchus browni 0010000110000--- 0010000110000---
11000010000100001000000000000000000000000000010000-------0-00200--
00000001010000000110100000---0--00-0-001---

Prolacerta broomi 0000000100000---1100000-
000000000000000000001110000000000101000000-------0-00000--0010-
000010010000101100l0?---1--00-?0?00---

Protorosaurus speneri 0000000000000---0?00000-00000000[01]?10001???1-
???0?????0010000??00-------0-00010--???1-010010?10?00-?1??00??---?--0?-?0?0?---

Rhynchosaurus articeps
0010100111001200011000110100100011110001010 1?1010000110111112110????000?
????20???????-00[01]0?0??01000001000?00??0??10??0001???

Stenaulorhynchus stockleyi 0010110111101200?110100-
11011000111101010101111101001100111211011100001110020111100000000000100
1010?01111111101101100000000

Supradapedon
????????????11?1???112????0-
21120101???????????????????1????????????101000??1?????001

Teyumbaita sulcognathus 11110111111111110011110-0111111111111011111-
0111110111111112111-
1100120101121111001111??11??11????01010110110010101010101

4 – SYNAPOMORPHY LIST

Synapomorphies of 11 most parsimonious tree recovered in the present analysis.

Rhynchosauria:
All trees:
Char. 3: 0 --> 1
Char. 9: 0 --> 1
Char. 23: 0 --> 1
Char. 28: 0 --> 1
Char. 60: 0 --> 1
Char. 76: 0 --> 2
Char. 93: 1 --> 0
Char. 100: 1 -->0

Howesia + *Eohyosaurus* + Rhynchosauridae:
All trees:
Char. 10: 0 --> 1
Char. 34: 0 --> 1
Char. 59: 0 --> 1
Char. 72: 0 --> 1

Eohyosaurus + Rhynchosauridae:
All trees:
Char. 29: 0 --> 1

Rhynchosauridae:
All trees:
Char. 5: 0 --> 1
Char. 17: 1 --> 0
Char. 19: 0 --> 1
Char. 36: 0 --> 1
Char. 40: 0 --> 1
Char. 48: 0 --> 1
Char. 60: 1 --> 2
Char. 61: 0 --> 1

Bentonyx + *Langeronyx* + *Fodonyx* + Hyperodapedontinae:+ Stenaulorhynchinae:
Char. 11: 0 --> 1
Char. 21: 0 --> 1
Char. 50: 0 --> 1
Char. 97: 0 --> 1
Char. 104: 0 --> 1
Char. 107: 0 --> 1

Bentonyx + *Langeronyx* + *Fodonyx* + Hyperodapedontinae:
All trees:
Char. 12: 0 --> 1
Char. 14: 2 --> 0
Char. 63: 0 --> 1

Langeronyx + *Fodonyx* + Hyperodapedontinae:
All trees:
Char. 28: 1 --> 0
Char. 101: 1 --> 0
Char. 111: 1 --> 0

Fodonyx + Hyperodapedontinae:
All trees:
Char. 109: 0 --> 1

Hyperodapedontinae:
All trees:
Char. 4: 0 --> 1
Char. 5: 1 --> 0
Char. 7: 0 --> 1
Char. 14: 0 --> 1
Char. 16: 0 --> 1
Char. 39: 0 --> 1
Char. 41: 0 --> 1
Char. 55: 0 --> 1
Char. 68: 0 --> 1
Char. 74: 0 --> 1
Char. 75: 0 --> 1
Char. 77: 0 --> 1
Some trees:
Char. 67: 0 --> 0/1
Char. 72: 0 --> 0/1

Hyperodapedon spp. + *Teyumbaita* + *Supradapedon*
All trees:
Char. 69: 0 --> 1
Char. 71: 1 --> 0
Some trees:
Char. 30: 0 --> 1
Char. 31: 0 --> 1
Char. 52: 0 --> 1
Char. 85: 0 --> 1
Char. 121: 0 --> 0/1
Char. 123: 0 --> 0/1

Hyperodapedon mariensis + *Hyperodapedon huxleyi* + *Hyperodapedon* Zimbabwe + *Hyperodapedon sanjuanensis* + *Hyperodapedon* Nova Scotia + *Hyperodapedon* Wyoming:
All trees:
Char. 76: 2 --> 1
Some trees:
Char. 79: 1 --> 0
Char. 120: 0 --> 1

Teyumbaita + *Hyperodapedon huenei*:
All trees:
Char. 65: 0 --> 1
Some trees:
Char. 15: 0 --> 1
Char. 23: 1 --> 0
Char. 67: 1 --> 0
Char. 106: 0 --> 1
Char. 119: 0 --> 1
Char. 123: 0 --> 1

Hyperodapedon sanjuanensis + *Hyperodapedon* Nova Scotia + *Hyperodapedon* Wyoming
All trees:
Char. 76: 2 --> 0
Char. 78: 1 --> 0
Char. 109: 1 --> 0

Hyperodapedon Nova Scotia + *Hyperodapedon* Wyoming
All trees:
Char. 71: 0 --> 1
Char. 115: 1 --> 0

Stenaulorhynchinae:
All trees:
Char. 73: 0 --> 1
Char. 110: 0 --> 1

Brasinorhynchus + *Stenaulorhynchus*+ *Mesodapedon*:
All trees:
Char. 65: 0 --> 1
Char. 108: 0 --> 1

5 – BOOTSTRAP AND BREMER-SUPORT VALUES

Clade support values provided in the figures below:

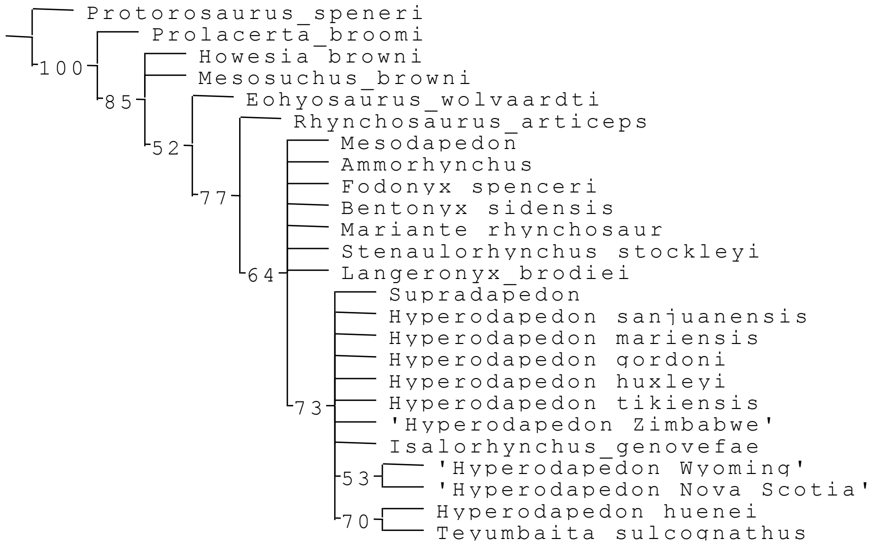

Fig. S1: Absolute bootstrap tree (1.000 replicates) showing group frequencies (cut = 50%)

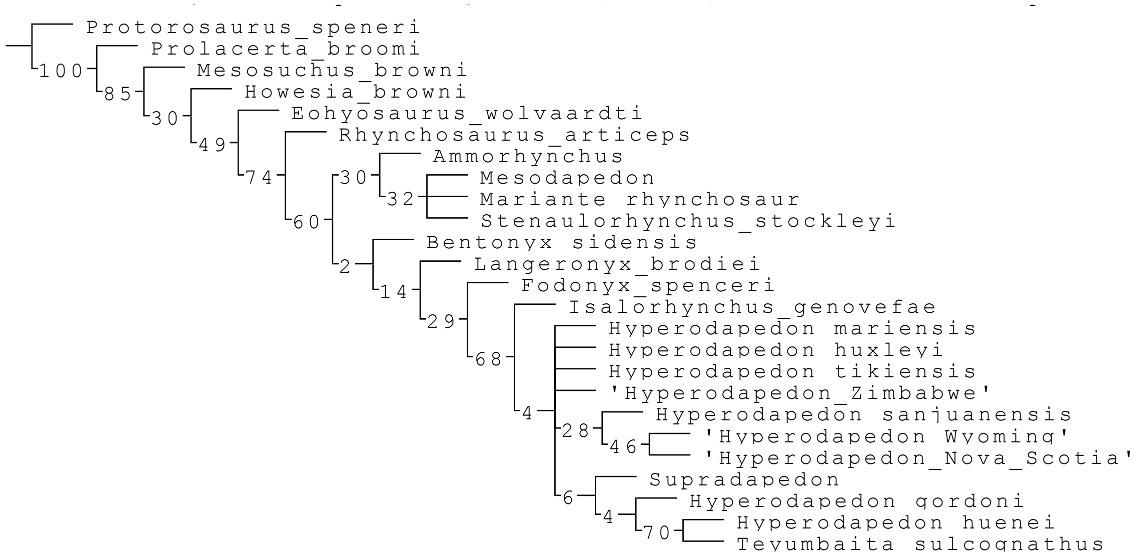

Fig. S2: Group present/Contradicted bootstrap tree (1.000 replicates) showing group frequencies (cut = 1)

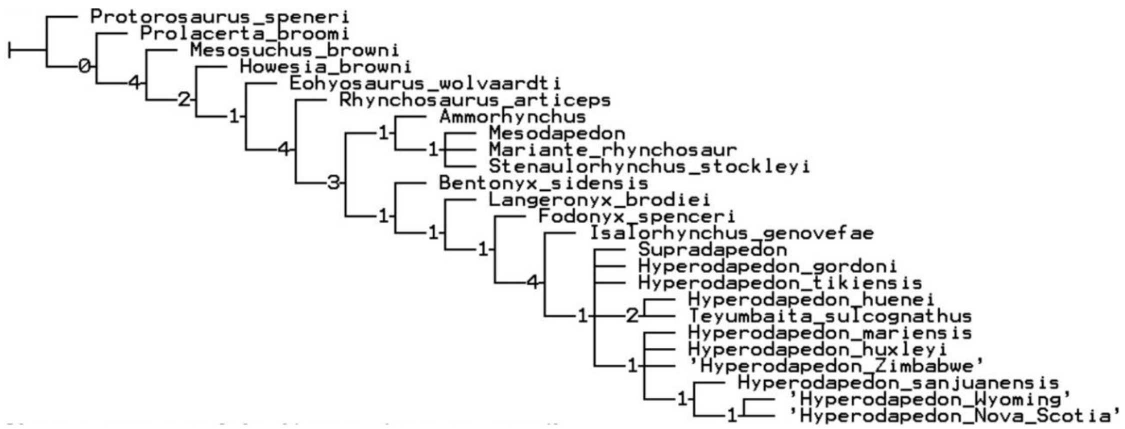

Fig. S3: Decay (Bremer support) values for each clade of the Strict Consensus Tree
